# Geological mapping of the Adrar Souttouf mafic complex in the Northern West African Craton using remote sensing and geophysical data

**DOI:** 10.1038/s41598-026-54473-5

**Published:** 2026-05-27

**Authors:** El Houcine El Haous, Abdelilah Fekkak, Youssef Houali, Said Haidatte, Abdelkrim Bouasria, Mohammed Boumehdi, Lahsen Achkouch, Felix Genske, Faouziya Haissen, Abdellatif Jouhari, Ilyasse Berrada, Nour Eddine Berkat, Abdellatif Errami, Othman Sadki, Ali Shebl

**Affiliations:** 1 Faculty of Sciences, Earth Sciences Department, Chouaib Doukkali University, El Jadida, Morocco; 2 National Office of Hydrocarbons and Mines, Rabat, Morocco; 3 Institute for Mineralogy, University of Münster, Münster, Germany; 4 Faculty of Sciences, Earth Sciences Department, Hassan II Ben Msik University, Casablanca, Morocco; 5 ESEF, Ibno Zohr University, Agadir, Morocco; 6 Geology Department, Faculty of Science, Tanta University, Tanta, 31527 Egypt; 7 Mineralogy and Geology Department, Debrecen University, Debrecen, 4032 Hungary

**Keywords:** Sentinel-2, Landsat OLI, Airborne gamma-ray spectrometry, Lithological mapping, Oulad Dlim massif, Environmental sciences, Solid Earth sciences

## Abstract

Geological mapping of the Adrar Souttouf Mafic Complex (ASMC) is constrained by its remote arid mountainous setting, where logistical limitations hinder traditional field surveys; however, advances in satellite data have provided powerful, cost-effective tools for high-resolution lithological and structural mapping. This research presents the first synergistic use of Sentinel-1 radar, Sentinel-2 and Landsat-8 optical imagery, and airborne gamma-ray spectrometry data to decipher the main structural and lithological characteristics of the Adrar Souttouf mafic complex within Morocco’s Oulad Dlim Massif. To achieve this, image processing—including color composites, band ratios, Principal Component Analysis (PCA), and radiometric terrain correction (RTC) —was employed for lithological mapping at regional and local scales. These spectral results were integrated with airborne gamma-ray spectrometry (K%, Th ppm, U ppm), providing the geochemical constraints necessary to differentiate lithological units and update existing geological maps. The study revealed that the highly complex geology with a variety of facies. These facies can be classified into three main categories: (i) (minor) ultramafic cumulates (ii) massive isotropic gabbros that represent magmatic intrusions that correspond to interconnected laccolites through a network of feeding dykes, and (iii) evolved silica-rich facies corresponding to charnockites, that are found either in undeformed massifs, or in elliptical bodies. The latter are situated under the broad gneissified massif of Hassi Bougaffa. All these magmatic facies were emplaced within a strongly deformed gabbroic body and are structurally controlled by fault systems. The most significant faults are oriented E-W and NNE-SSW. The result validation by fieldwork aided by remote sensing and gamma-ray spectrometry maps have clearly helped in better distinguishing between the various lithological units. This led to update the litho-structural map of the study area.

## Introduction

Accurate geological mapping is essential to understanding the lithological and structural evolution of the Earth’s crust. While traditional field mapping remains the cornerstone of geological inquiry in frontier regions, its execution is often constrained by significant logistical overhead, protracted timelines, and the hazards of remote accessibility. To overcome these restrictions, combining remote sensing and geophysical techniques has emerged as an effective strategy for improving lithological discrimination and geological interpretation^1–6^.

Remote sensing data provides critical spectral insights that reflect the mineralogical and chemical composition of surface materials. As documented by several studies utilizing multispectral platforms—including Landsat, Sentinel-1 and 2, and ASTER^7–12^—extracting this data requires specialized image processing. Geologists typically employ enhancement techniques such as Principal Component Analysis (PCA), decorrelation stretching, and band ratios to differentiate lithological units and identify structural features like folds and faults^13–19^.

Simultaneously, the natural radioactivity of rocks and soils is measured by gamma-ray spectrometry data (radiometric data), mainly from uranium (U), thorium (Th), and potassium (K) isotopes. The distribution of these radioelements is determined by weathering intensity, alteration processes, and lithological composition^6,20–22^. In areas with little field exposure, radiometric maps and derived ratio maps like Th/K, U/K, and Th/U have been especially useful for defining lithological boundaries and improving geological mapping^23–28^.

The integration of remote sensing and gamma-ray spectrometric provides a solid basis for improving geological interpretation^6^. The combination of remote sensing spectral features with geophysical data from radiometric surveys can lead to improved field observations and more accurate lithological mapping^29–31^.

In this study, we combined these data with aim of refining lithological and structural mapping in the Adrar Souttouf Mafic Complex (ASMC), which is a part of the Northern West African Craton. This integrated approach is expected to improve understanding of spatial lithological relationships and produce a more comprehensive geological description of the terrain.

The objectives of this study are to: i) perform a multi-sensor remote sensing interpretation of Sentinel and Landsat images to extract the various geotectonic units (lithology and lineaments), (ii) analyze the spatial relationships between lithologies and fault systems; (iii) refine and update existing geological maps by identifying previously unmapped or poorly constrained outcrops, thereby extending the work of^8^,and (iv) produce a revised geological map that constitutes the first remote-sensing-based, integrated litho-structural framework of the ASMC, offering new insights into its internal architecture and tectonic organization. By delivering the first gamma-ray and remote-sensing-integrated map of the ASMC, this work constitutes an original contribution and a definitive technical benchmark for future geological exploration of the studied terrain.

## Geological setting

**Fig. 1:**
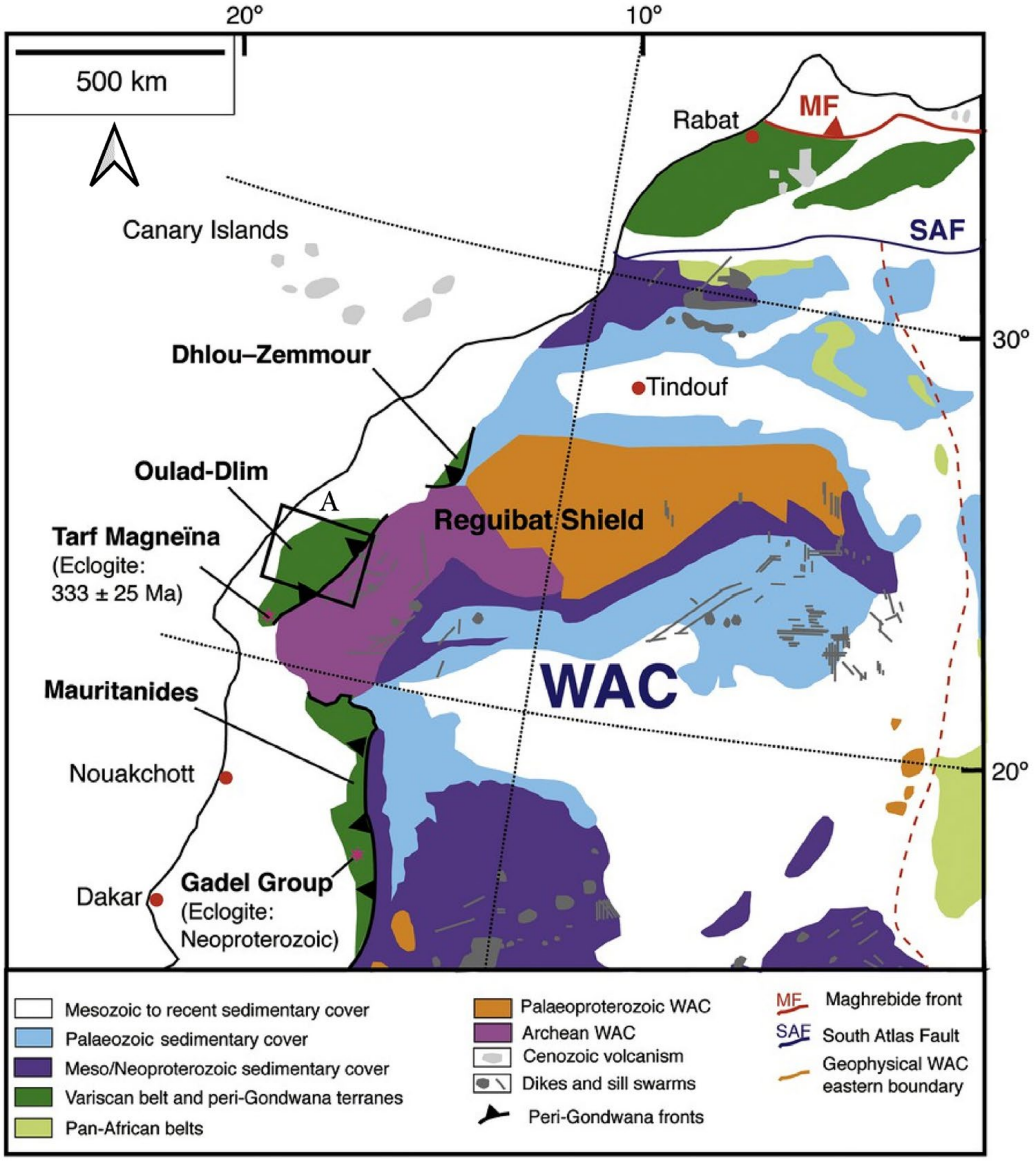
Simplified geological map of the West African Craton and its location within the African continent^41–43^. The square labelled as A is shown in Figs. 2 and 3.

**Fig. 2:**
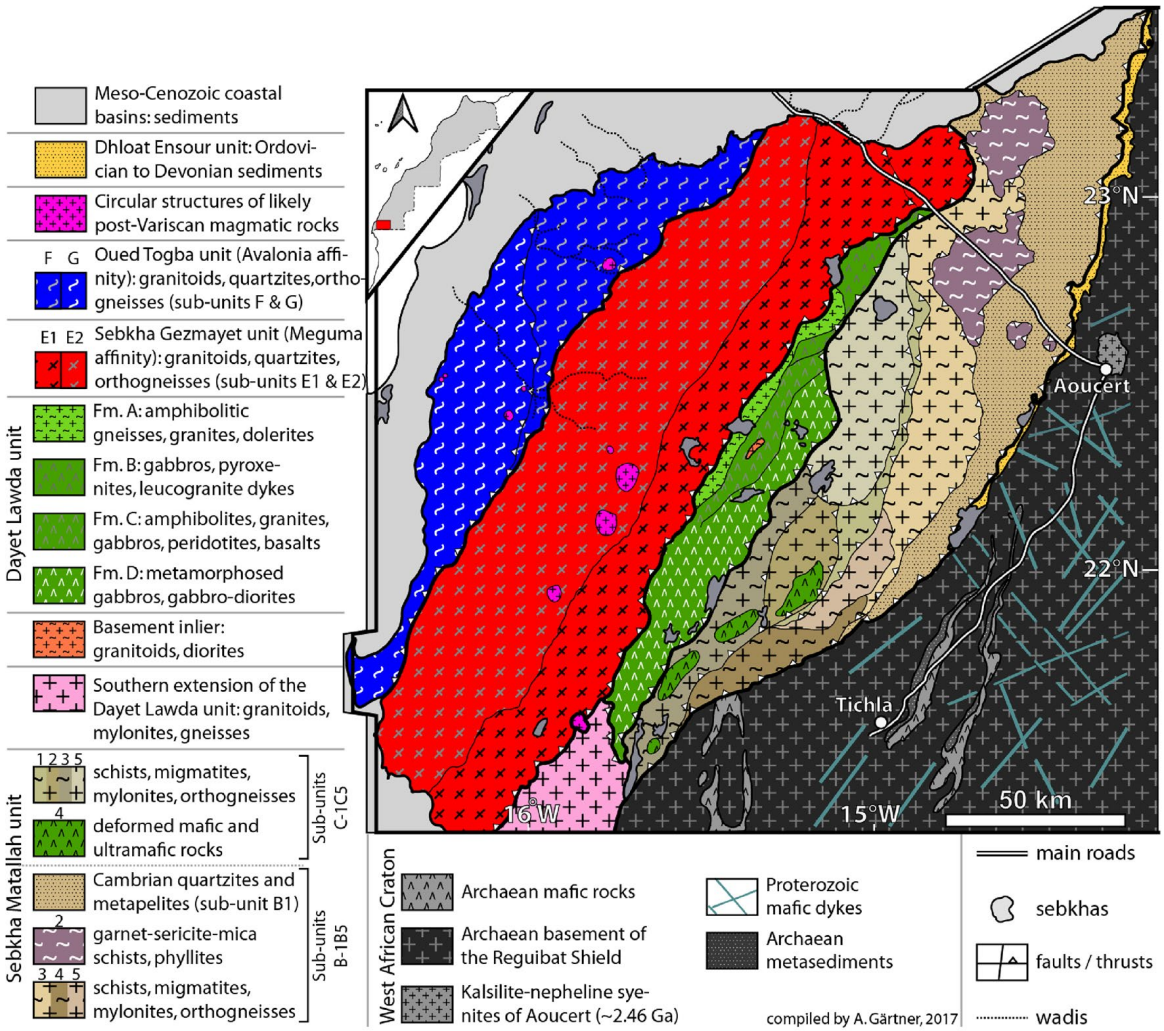
Lithological map and main metamorphic units of Oulad Dlim massif after Gärtner et al.^39^.

**Fig. 3:**
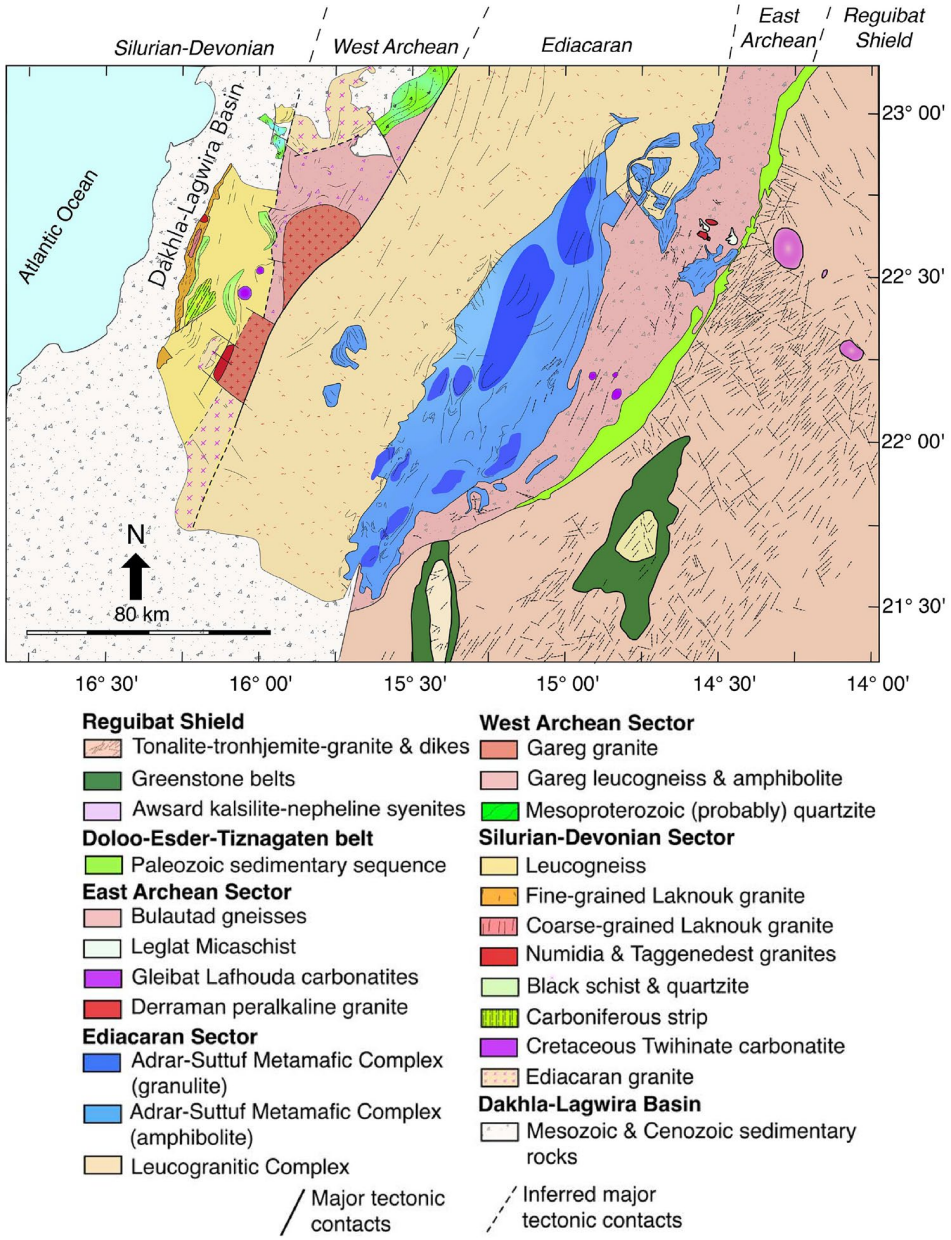
Geological scheme and sectors of the Oulad Dlim^40^.

The Oulad Dlim Massif, according to the first model, which was proposed by Sougy^32–34^, Marchand et al.^35^, and Bronner et al.^36^ and later supported by Villeneuve et al.^37,38^ and Gärtner et al.^39^, is a klippen-type structure located in the southernmost part of Morocco, and is composed of four metamorphic thrust units that sit on top of the Palaeozoic cover to the east (Fig. 1). Gärtner et al.^39^, describe this massif as being composed of four major units that are arranged from west to east (Fig. 2): The Oued Togba Unit, the Sebkhat Gezmayet Unit, the Dayet Lawda Unit, and the Sebkha Maatallah Unit. These units are obviously divided by thrust faults. The Archean Reguibat Ridge and the Oulad Dlim Massif are separated by the Palaeozoic Dhloat Ensour Unit. In contrast, the second interpretation proposed by Bea et al.^40^ considers the earlier model to be overly speculative and proposes a simpler geological framework. In line with this theory (Fig. 3), the study area comprises a central Ediacaran domain flanked by eastern and western Archean domains. The Ediacaran domain is subdivided into two complexes: a felsic complex and a mafic complex.

Except for the Tagennadest Fault and the tectonic contact that separates the eastern Archean domain from the Reguibat Shield, Bea et al.^40^ did not find any compelling evidence for the existence of overlapping contacts within or between the various domains in their proposed structural map. Thus, the four major domains that span the massif from west to east were determined to be the Ediacaran domain (which includes a felsic and a mafic complex), the western Archean domain, the eastern Archean domain, and the Silurian–Devonian domain. The primary source of disagreement between these two models is how the Adrar Souttouf mafic complex, the focus of this study, is interpreted geologically. According to the Klippen structure model, the complex was formed by the closure of three successive oceans: The Rheic Ocean, the Iapetus Ocean, and the West African Neoproterozoic Ocean. However, the synformal model interprets the complex as evidence of an intracontinental rift that runs across Africa.

According to^44^, the first discovery of eclogites in the Lamlaga area of Oulad Dlim massif provide clear evidence of subduction-related metamorphism within the peri-cratonic domain of the West African Craton. They originated from a mafic protolith dated at 584 ± 6 Ma, contemporaneous with widespread Neoproterozoic bimodal magmatism in the region.

**Fig. 4:**
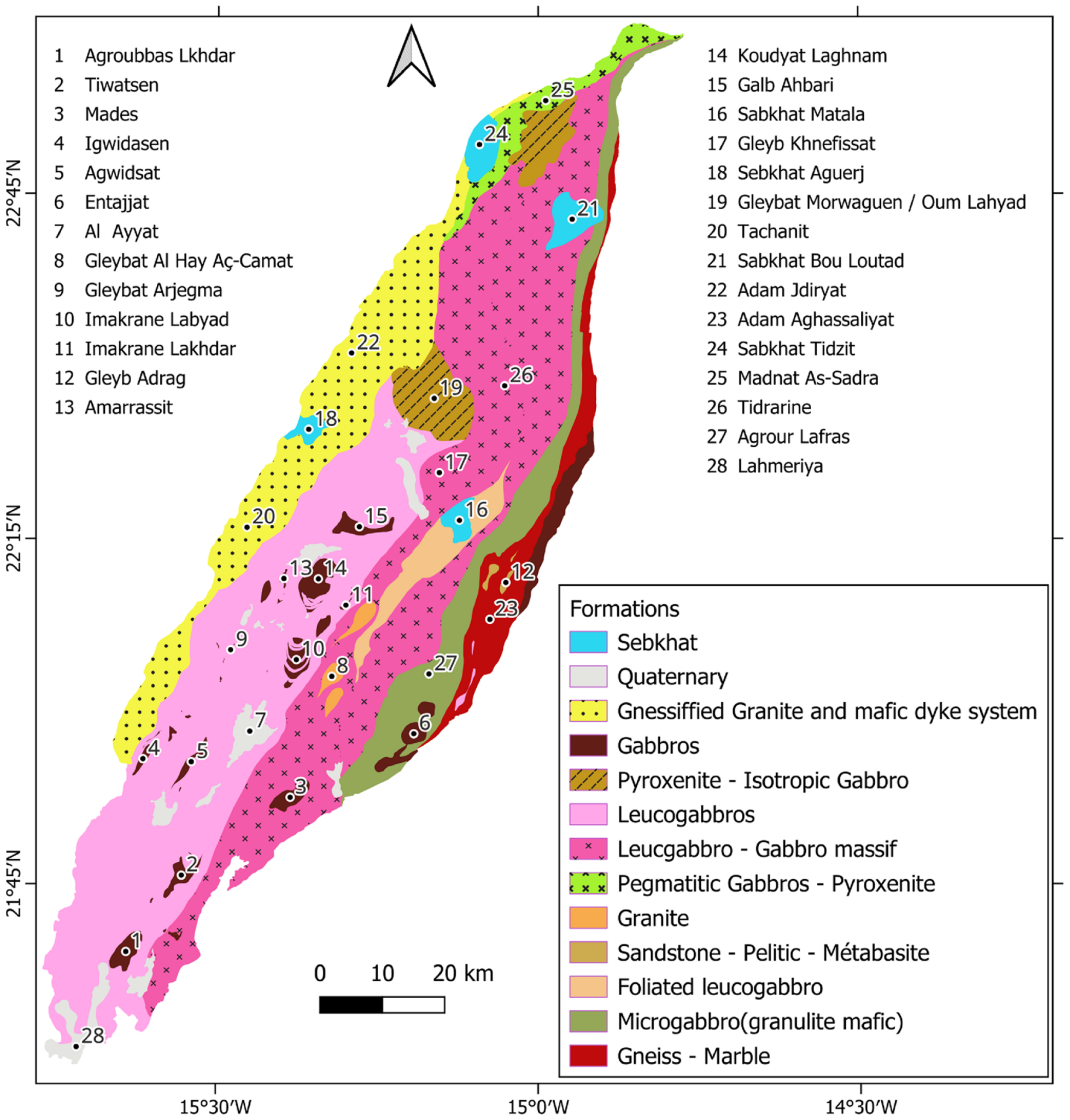
Lithological map of ASMC (Oulad Dlim Massif, Southernmost Morocco)^8^.

A recent study carried out by El-Haous et al.^8,13^ for producing a more detailed map (Fig. 4), suggests that the Adrar Souttouf mafic complex is mainly composed of mafic rocks (foliated leucogabbro, isotropic gabbros, pegmatitic gabbros and pyroxenites) with a significant dyke field located to the west in gneissified granites. The Al Ghassaliyat subunit, which consists mainly of gneisses and marbles, limits the complex to the east. Fekkak et al.^45,46^ confirm the detailed geological framework of the study area based on national geological mapping of the Oulad Dlim Massif.

## Materials and methods

### Remote sensing data and field work

The study area is located in southern Morocco, around 80 km west of the town of Awserd, and is elliptical in shape, oriented NNE-SSW, with maximum dimensions of around 186 × 52 km. covering an area of 6176 km^2^, characterized by the absence of vegetation cover due to the arid climate of the region, which is beneficial when applying processing to the satellite images.

#### Satellite data

In this work, data from three different satellites were used as shown in Table 1:

**Table 1:** Main characteristics of the three different sources of the used satellite data.

Satellite	Sentinel-1B	Sentinel-2A	Landsat-8
Used sensor	C-SAR (Radar)	MSI (Multispectral)	OLI (Multispectral)
Spatial resolution	5 × 20 m (IW mode)	Down to 10 m (VNIR)	Down to 15 m (PAN)
Swath width	250 km	290 km	185 km
Viewing angle	20° to 50° off-nadir	± 10.3° off-nadir	± 7.5° off-nadir
Coverage time	2016 to present	2015 to present	2013 to present

This research utilized multi-source satellite data, comprising Sentinel-1B, Sentinel-2A, and Landsat-8 images. The Sentinel-2A Level-2A and Landsat-8 Collection 2 Level-2 products signify processed datasets that relate to surface reflectance data, with atmospheric, radiometric, and geometric corrections already implemented^47,48^. Typically, Level-2 products signify satellite data that has been processed from raw Level-1 data to eliminate atmospheric influences and transform digital numbers into values that are physically significant and appropriate for immediate scientific examination^49,92^. In comparison, Sentinel-1B SAR data necessitated further preprocessing steps, such as radiometric calibration, speckle filtering, and terrain correction, before interpretation and analysis could occur^50,51^.

**Fig. 5:**
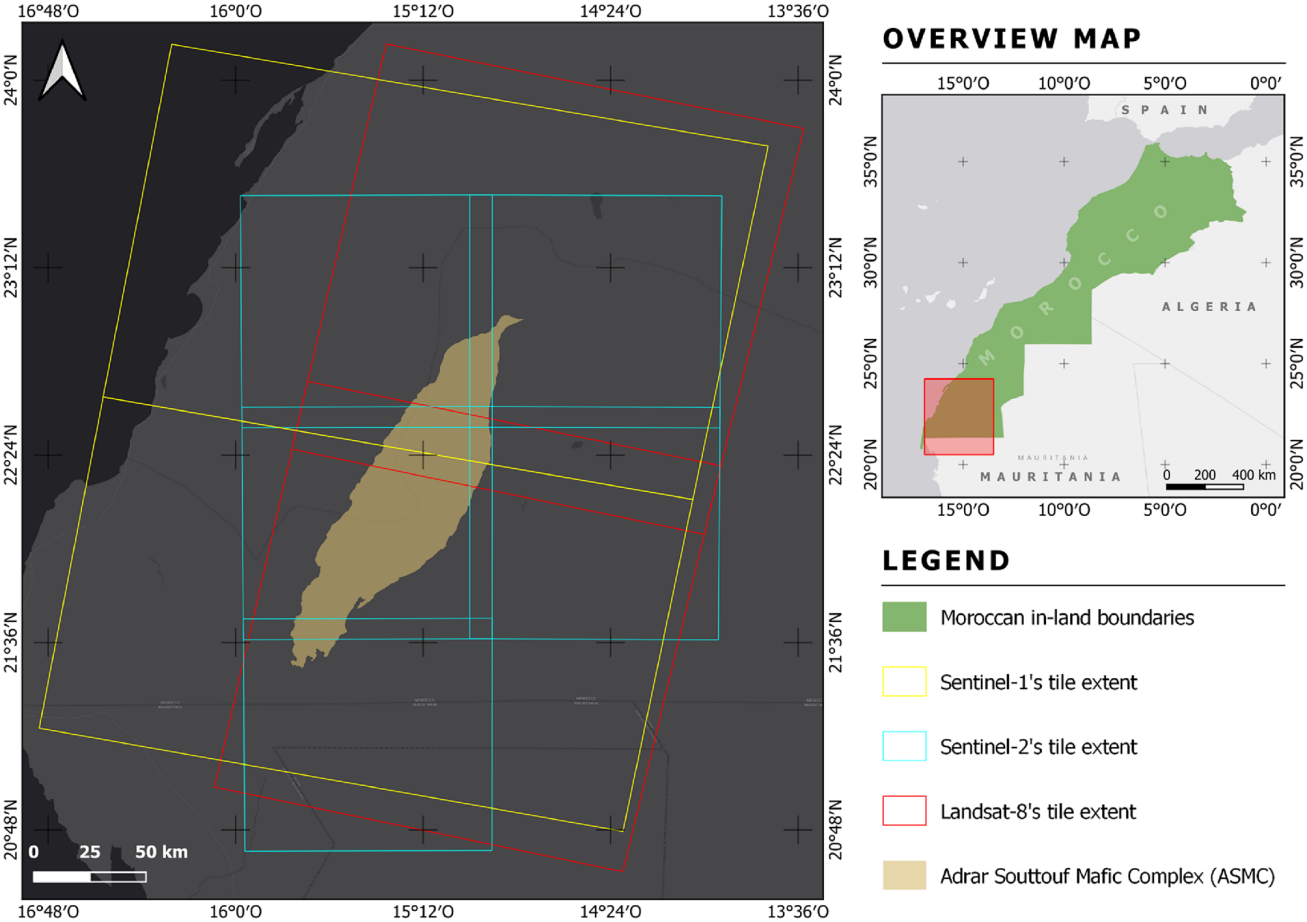
Footprints of the Sentinel-1B, Sentinel-2A and Landsat-8 tiles covering the ASMC.

Due to the satellites’ large swaths, the derived products cover perfectly the entire extent of the area of study within the same orbital pass which makes the mosaicking work more precise. This is very important in our context (structural and geological mapping) as any misalignment between the mosaic tiles could cause a false interpretation of the results. Figure 5 shows the extent of each satellite’s swath covering the ASMC.

Despite the finest spatial resolution that each satellite’s sensor can offer, we decided to adopt those corresponding to the majority of the bands of interest, which are 30 m for Landsat 8 and 20 m for Sentinel-2A (Table 2). The reason behind this is to prevent any loss of spectral information due to the resampling process. Table 3 enumerates the different downloaded products. The Landsat and Sentinel data were downloaded, respectively, from *earthexplorer.usgs.gov* and *browser.dataspace.copernicus.eu.*

**Table 2:** Comparison of landsat-8 and Sentinel-2 satellite bands.

Landsat 8 OLI			Sentinel-2 MSI		
Bands	Wavelength range (nm)	Res. (m)	Bands	Wavelength range (nm)	Res. (m)
1 Coastal aerosol	433—453	30	1 Coastal aerosol	433—453	60
2 Blue	450—515	30	2 Blue	458—523	10
3 Green	525—600	30	3 Green	543—578	10
4 Red	630—680	30	4 Red	650—680	10
			5 Red edge 1	698—713	20
			6 Red edge 2	733—748	20
			7 Red edge 3	773—793	20
5 Near infrared	845—885	30	8 Near infrared	785—900	10
			8a Near Infrared Narrow	855—875	20
			9 Water Vapour	935—955	60
9 SWIR / Cirrus	1360—1390	30	10 SWIR / Cirrus	1360—1390	60
6 Shortwave infrared 1	1560—1660	30	11 Shortwave infrared 1	1565—1655	20
7 Shortwave infrared 2	2100—2300	30	12 Shortwave infrared 2	2100—2280	20
8 Panchromatic	500—680	15

**Table 3:** List of the different used satellite products.

Mission	Sensor	Process level	Product identifier	Date
Landsat-8	OLI	L2SP	LC08_L2SP_**205044**_20211210_20211215_02_T1	15–12-2021
			LC08_L2SP_**205045**_20211210_20211215_02_T1	
Sentinel-2A	MSI	L2A	S2A_MSIL2A_20211223T113511_**N0301_R080_T28QDJ**_20211223T142742	23–12-2021
			S2A_MSIL2A_20211223T113511_**N0301_R080_T28QDK**_20211223T142742	
			S2A_MSIL2A_20211223T113511_**N0301_R080_T28QDL**_20211223T142742	
			S2A_MSIL2A_20211223T113511_**N0301_R080_T28QEK**_20211223T142742	
			S2A_MSIL2A_20211223T113511_**N0301_R080_T28QEL**_20211223T142742	
Sentinel-1B	C-SAR	GRD	S1B_IW_GRDH_1SDV_20190508T065524_20190508T065547_**016149_01E622_4657**	08–05-2019
			S1B_IW_GRDH_1SDV_20190508T065459_20190508T065524_**016149_01E622_BD8B**	

#### Airborne radiometric data

To characterize lithological variations and their structural organization within fault-controlled zones hosting intrusive dykes, the adopted methodology begins with the analysis and interpretation of radiometric data. These lithology-related structural constraints will then be used to reconstruct the tectono-lithological evolution of the massif.

An National Office of Hydrocarbons and Mines (ONHYM) commissioned fixed-wing airborne geophysical survey was flown by Fugro in 2002 over ~ 43,790 line-km at a nominal terrain clearance of 100 m. Production lines trended 115° with 500 m spacing, and control (tie) lines 25° at 5 km spacing. Navigation employed a NovAtel 3151R real-time DGPS with OmniStar differential corrections; all streams (radiometric, and navigation) were synchronized to the GPS 1-PPS signal. Airborne gamma-ray spectrometry was acquired contemporaneously using an Exploranium GR820-3 (MiniDAS) with a GPX 1024/256 NaI (Tl) detector package comprising a downward-looking 2,048 in^3^ (33.56 L) crystal and an upward-looking 256 in^3^ (4.2 L) crystal. Radiometric channels were sampled at 1.0 s, corresponding to ~ 75–80 m along-track spacing at survey speed. These parameters provide the spatial resolution required for robust structural mapping from radiometric gradients.

#### Field work

Three planned field trips were undertaken. The initial objectives were to locate the study area and create a synthetic geological cross-section of every geological formation encountered during image processing. After the results were processed and synthesized, a second mission was planned to confirm the final geological map that was finally produced. A third field mission was subsequently carried out to address the gaps identified during this validation phase and to refine the final geological map interpretation.

### Methods

In this study, we used different type of data (optical, radar, and radiometric imagery and field observations) in order to get a maximum information that help in mapping the structural and geological features of the studied area.

**Fig. 6:**
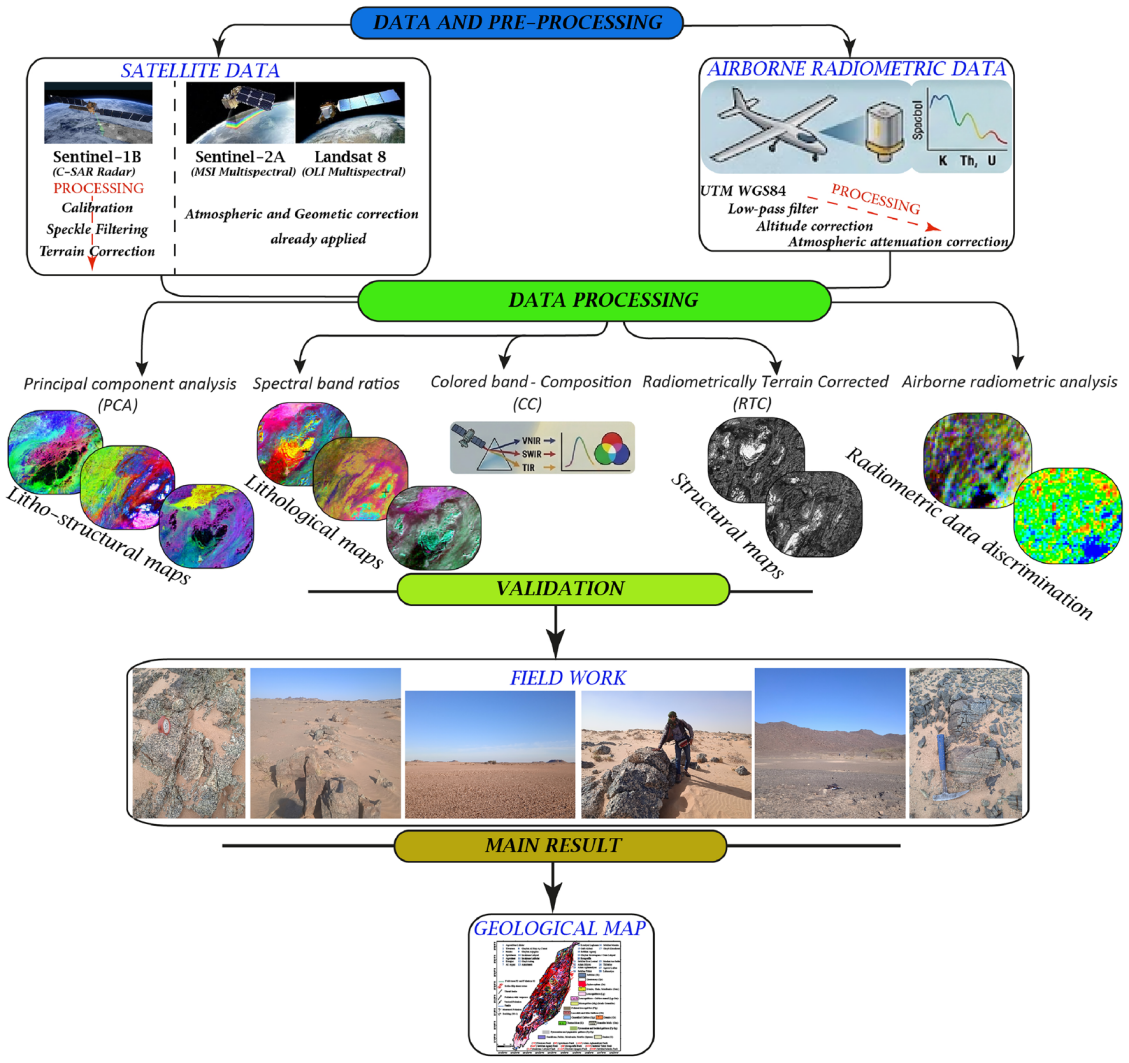
Flowchart showing the adopted methodology utilized in the current study.

Methods such as the principal component analysis (PCA), spectral bands ratio (BR) and colored band composition (CC), were used to process optical data, while the radar data were processed using radiometric terrain correction (RTC). Additionally, to improve the visualization of lithological variations and possibly the areas of rock alteration, a ratio, false-color composite image of the radioelements K%, Th ppm and U ppm was generated. A flowchart illustrating the methodology adopted in the present study is presented in Fig. 6. The processing of airborne gamma-ray spectrometry data and figure preparation were carried out in accordance with the authorization granted by ONHYM. Oasis Montaj software was used for radiometric data processing, while ArcGIS was employed for spatial analysis and map production.

#### Principal component analysis (PCA)

Principal Component Analysis (PCA) is a statistical method for projecting multidirectional data and reducing data dimension, applicable also for geological interpretation^52^. Selecting the three best bands for a color composite is made much easier with PCA, an effective technique for spectral enhancement and information extraction^53–56^. This method, initially introduced by Hotelling in 1933, is based on the notion that the data being examined are highly correlated. The objective of this analysis is to condense the data from several strips into a limited number of components. In our study, we used this technique to compress the data scattered across the spectral bands of the satellite images.

#### Spectral band ratios

To improve geological mapping and lithological unit discrimination, several spectral band ratios were created. Band ratioing is a popular remote sensing method that highlights differences in surface mineralogy and rock composition while reducing the impact of topography, shadow, and illumination^5,57^. Certain spectral responses of rock-forming minerals are highlighted in the resulting image by dividing one spectral band by another. These responses are crucial indicators in geological interpretation^58,59^.

#### Colored band-composition (CC)

The process of combining colored bands (CC) is one of the most basic methods used in multispectral remote sensing that allows for improved image interpretation through the combination of selected bands in the red, green, and blue (RGB) channels. Each band is based on reflectance in a certain wavelength range, and their combination helps identify features on the Earth’s surface that would be difficult to detect using individual bands. In true color composite images, visible light bands are used to represent colors as we would see them in reality, while false color composites include invisible wavelengths, such as near-infrared (NIR) and short-wave infrared (SWIR), to highlight different land cover types. It relies on the unique spectral properties of the material, and therefore, proper band selection can increase contrast between vegetation, soils, water, and urban surfaces^60–62^.

#### Radiometrically terrain corrected products (RTC)

Sentinel-1 Radiometrically Terrain Corrected (RTC) images refer to SAR data aimed at eliminating geometric and radiometric distortions caused by the influence of terrain features. The process of generating RTC data involves radiometric calibration to transform backscatter to sigma nought (σ⁰), beta nought (β⁰), or gamma nought (γ⁰) terrain corrected (RTC) images refer to SAR data aimed at eliminating geometric and radiometric distortions caused by the influence of terrain features. The process of generating RTC data involves radiometric calibration to transformtions and observation conditions. RTC images are geocoded and reprojected into a cartographic projection, which increases opportunities for combining them with other datasets. RTC Sentinel-1 images are widely used in land cover mapping, soil moisture assessment, and detecting changes due to their high sensitivity to surface roughness and electrical characteristics. In addition, RTC images are processed with speckle reduction filters to eliminate SAR interference. Hence, RTC images represent analysis-ready SAR data for advanced geospatial research^49,63,64^.

#### Airborne radiometric data

Airborne gamma-ray spectrometry was used to map the spatial distribution of K%, eU, and eTh as proxies for lithology and alteration^24,25,27,65,^^66^. These radioelements vary according to crustal composition and their gamma emissions allow discrimination of lithological units and alteration zones^24,25,27,67–69^.

Before being interpreted, the radiometric data were georeferenced and projected into the UTM WGS84 coordinate system to ensure spatial consistency with other geospatial datasets, such as geological and remote sensing data. A low-pass filter was applied to remove noise and non-geological short-wavelength anomalies, which mainly correspond to random statistical noise, striping effects, and minor sensor-related fluctuations rather than true geological signals^26^, and standard corrections for altitude and atmospheric attenuation were performed^22,70^.

Processed grids of K (%), eU (ppm), and eTh (ppm) were then used to generate ratio maps, particularly K/eTh, to enhance lithological discrimination and detect hydrothermal alteration. High K/eTh values indicate potassium enrichment related to felsic lithologies or potassic alteration, whereas low values reflect mafic rocks or leaching/weathering processes^22,24,25,67,71–73^.

Additionally, a false-color composite of K%, eTh, and eU was produced to enhance visualization of lithological contrasts and alteration zones^27,67,69,74–76^. This composite successfully draws attention to localized enrichment patterns that could be connected to alteration halos or structurally controlled mineralization, as well as distinctions between the felsic and mafic domains.

## Results

### Lithological mapping

#### True color composition

**Fig. 7:**
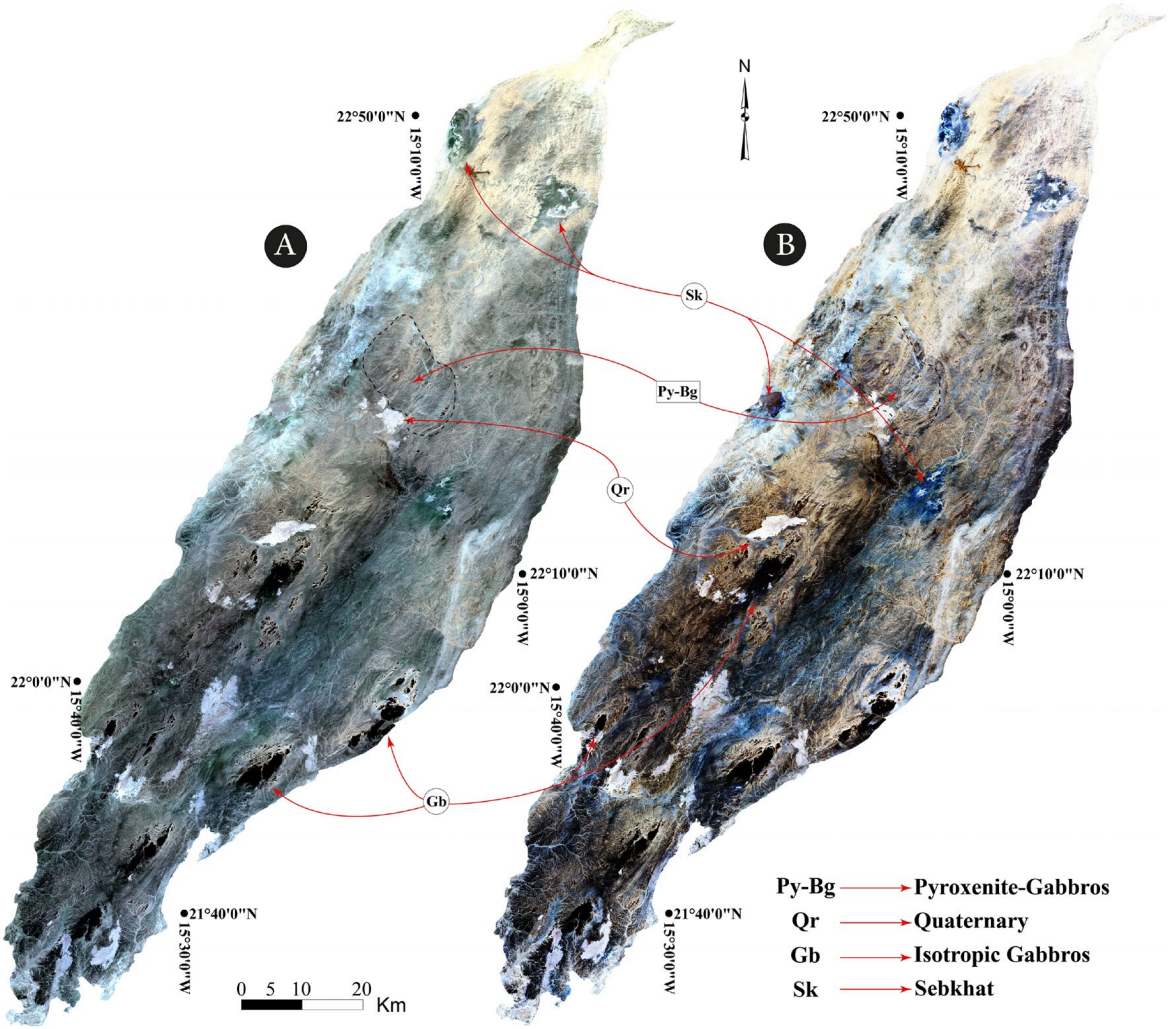
True colored composition 4–3-2 as RGB of Landsat 8 OLI (**A**) and Sentinel-2A (**B**) image bands.

Geological formations can be seen in their natural colors, which are 4–3-2 for Landsat 8 OLI and Sentinel-2A images, which is due to the application of the most important color compositions in RGB order. Figure 7A presents Landsat 8 OLI images displays Quaternary terrains in snow white, significant Gabbro outcrops in black, Sebkhat in greenish, and the apparent Pyroxenite alternation unit in sky blue with isotropic massive Gabbro in brown.

With still very good discrimination (Fig. 7B) of the Gabbro appearing in black, the Sebkhat in blue to dark blue, the Pyroxenite alternation unit in sky blue with isotropic massive Gabbro in brown, and the Quaternary terrains in whitish, the composition chosen in the VNIR bands for Sentinel-2A images is comparatively much more significant than those obtained from Landsat 8 OLI.

#### Principal component analysis (PCA)

**Fig. 8:**
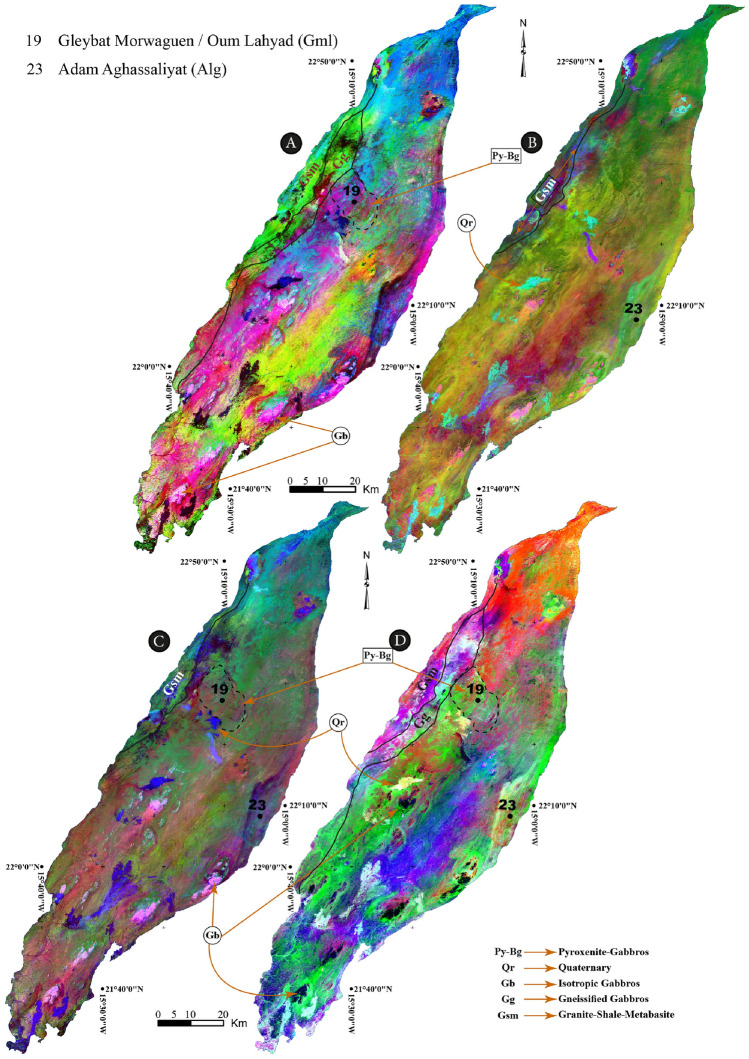
Principal component analysis (PCA) canals in RGB of Landsat 8 OLI. **A**). PCA-7,5,2. **B**). PCA-5,6,2. **C**). PCA-7,6,2. **D**).PCA-7,4,2.

**Fig. 9:**
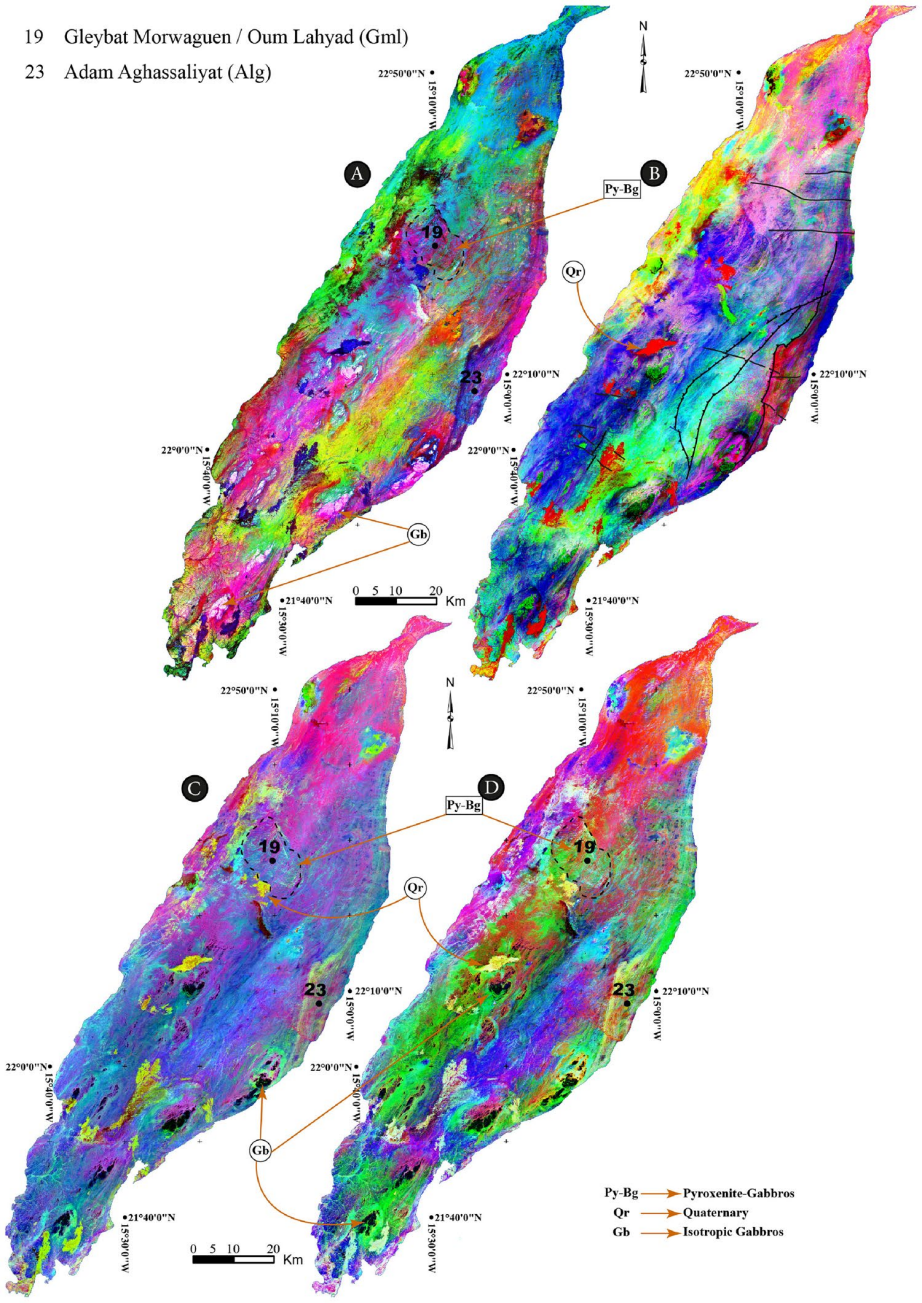
Principal component analysis (PCA) canals in RGB of Sentinel-2A. **A**). PCs-12,8,2. **B**). PCs-8,11,12. **C**). PCs-12,11,2. **D**). PCs-12,4,2.

Compared to the other principal component analyses, which yield highly satisfactory for both images used, the VNIR-SWIR bands compositions for the and Sentinel 2A PCs (12–11-2 in RGB) (Fig. 7C) and Landsat 8 OLI PCs (5–6-7 in RGB) (Fig. 8B) are comparatively less significant. These composites allow a clear discrimination between mafic rocks (Isotropic Gabbros) and Quaternary terrains are easily distinguished in this color composite, which was produced using various RGB filters (Fig. 9A-D for Sentinel 2A and Fig. 8A-D for Landsat OLI 8).

The false-color composites derived from principal components (PCs) enable the identification of nearly all lithological units. Distinct units include the massive Pyroxenite-Gabbro alternations of Gleybat Morwaguen and Gleyb Oum Lhyad, as well as the Adam Alghassaliyat unit composed of Garnet-Marble-Amphibolite Gneisses.

The Sentinel 2A PCs (8, 11, 12 in RGB) provided improved visualization of various ground deformations, particularly fault zones (Fig. 8B). In addition, the Sentinel 2A PCs (12, 8, 2 in RGB) composite and the Landsat 8 OLI PCs (7, 4, 2 in RGB) show excellent lithological discriminations. In these composites, gneissified granite at the eastern margin of the massif appears in magenta, while adjacent alternations of gneissic gabbro, schist and metabasite are displayed in light purple, highlighting sharp lithological contrasts.

#### Band ratio

In the current lithological discrimination, color band ratios (BRs) are much more effective than products based on principal components because they make direct use of the spectral behavior of rock-forming minerals in the VNIR–SWIR domains. These ratios highlight diagnostic absorption features related to mafic minerals, ferromagnesian silicates, carbonates and hydrated phases, while minimizing the impact of illumination and topography.

**Fig. 10:**
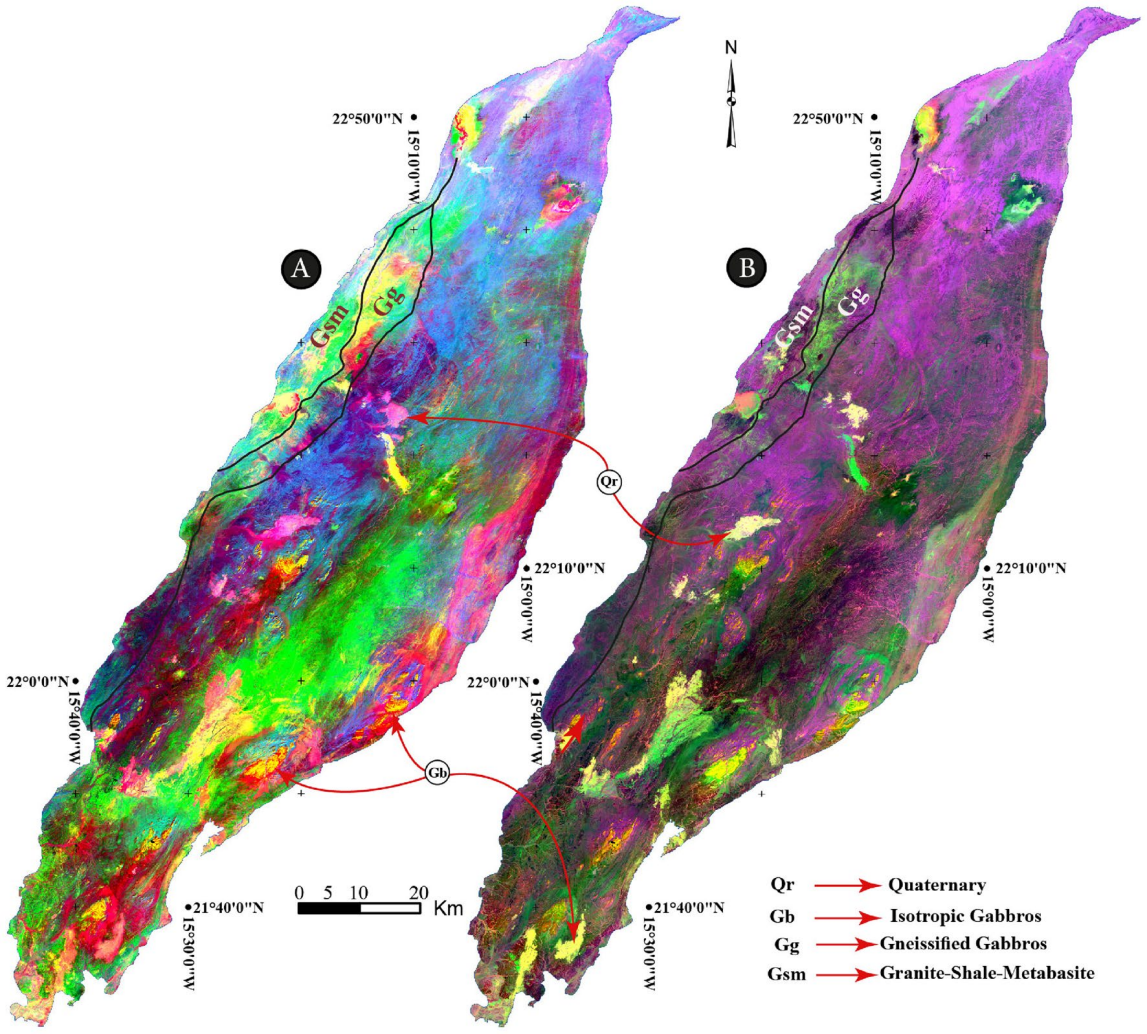
Combination of Landsat 8 OLI band ration (BR) in RGB: **A**). BRs (Hydrothermal alteration): 4/2, 6/5, 6/7 and **B**). BRs (rock unit identification): 4/2, 6/7, 5/4.

For, Landsat 8 OLI the BRs (4/2, 6/5, 6/7 in RGB) emphasize iron-oxide–rich surfaces (band 4), ferromagnesian minerals and clay-related absorptions (bands 6 and 7), allowing clear discrimination of isotropic gabbros from surrounding units^5^, Similarly BRs (4/2, 6/7, 5/4 in RGB)^77^ enhance contrasts between mafic bodies, gneissic gabbros, and Quaternary cover by combining visible iron-sensitive bands with SWIR bands sensitive to hydroxyl-bearing minerals (Fig. 10).

**Fig. 11:**
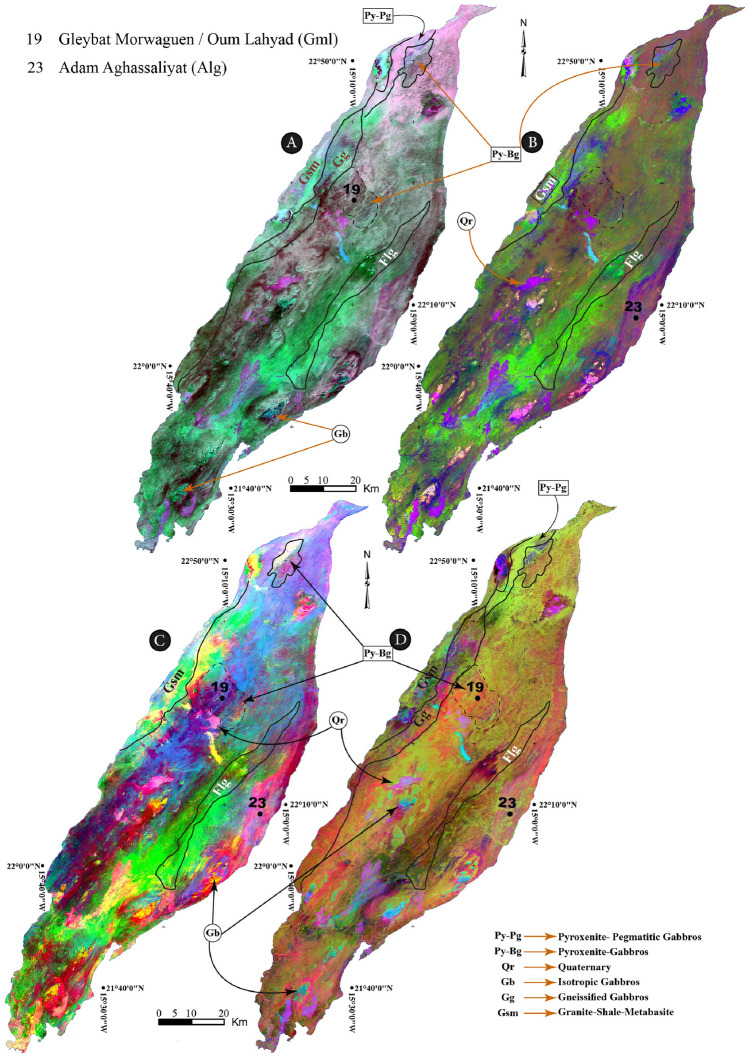
Combination of Sentinel-2A band ration (BR) in RGB: **A**). BRs: 11/12, 11/2, 11/8*4/8; **B**). BRs: 11/12 ,4/2, 8A/11; **C**). BRs: 12/8A, 8A/4, 11/12 and **D**). BRs: 3/6, 7/4, 5/2.

For Sentinel-2A, lithological discrimination is further improved using band ratios (11/12, 11/2, 11/8*4/8 in RGB), which enhance spectral contrasts related to variations in mafic mineral content and surface alteration. This approach combines SWIR bands, sensitive to Mg–Fe silicates, with VNIR bands that highlight iron oxides. It allows clear identification of lithological units, including isotropic gabbros and Quaternary deposits, as well as gneissified granite at the eastern end of the massif and the alternation of gneissic gabbro, schist, and metabasite units. This NNE–SSW-oriented unit appears in blue tones, reflecting the high shortwave infrared (SWIR) reflectance characteristic of gabbroic compositions. The BRs (11/12 ,4/2, 8A/11 in RGB) and BRs (12/8A, 8A/4, 11/12 in RGB) enhance the contrasts related to mineralogical composition and grain size to efficiently separate gabbroic and pyroxenitic units from gneissic and metamorphic lithologies. In addition, the BRs (3/6, 7/4, 5/2 in RGB) highlight subtle compositional variations within the massif. This enables better delineation of lithological boundaries, as well as improved characterization of internal heterogeneities, thereby enhancing the identification of mafic, metamorphic, and Quaternary units across the massif (Fig. 11). In the present study, band ratios were very effective in differentiating the different rock units and lithological mapping of the ASMC.

#### Structural lineaments

**Fig. 12:**
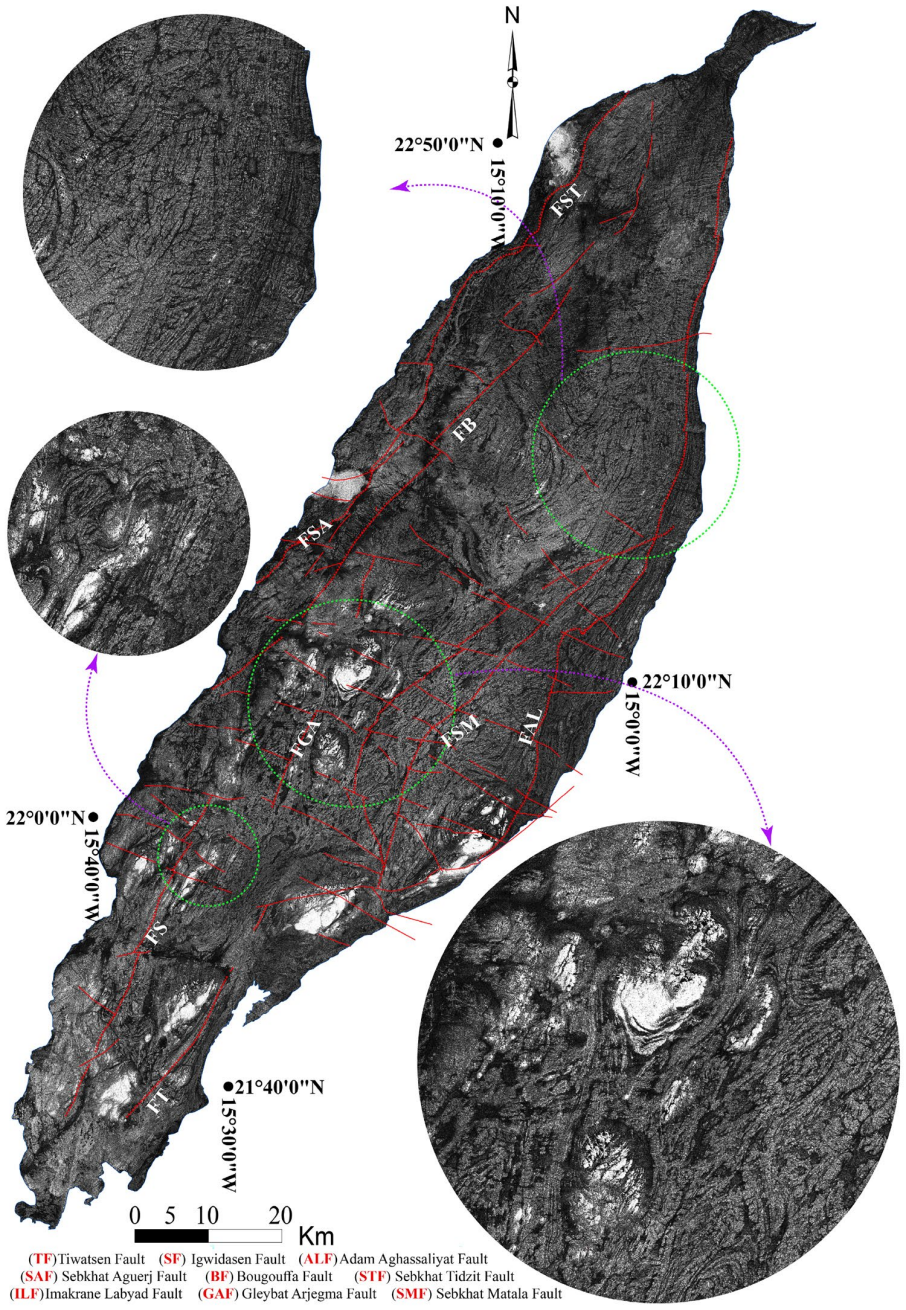
The radar image was processed using a radiometric terrain correction (RTC) which shows the faults.

Machine Learning-based results reported by^3,8,14^ reveal that Margin, PCA, and canonical correspondence analysis (CCA) approaches provide a better visualization of various ground deformations, especially faults. In the present study, RTC data (Fig. 12) were additionally used to enhance the detection and interpretation of structural lineaments and fault-related features. Structurally, the massif is characterized by prominent NE–SW to NNE–SSW lineaments that define its principal tectonic framework. These lineaments are concentrated within a large, well-developed shear zone that controlled both deformation and the spatial organization of the lithological units. This regional fault (Fig. 12) network includes the Imakrane Labyad Fault (ILF), Gleybat Arjegma Fault (GAF), Sabkhat Matala Fault (SMF), Tiwatsen Fault (TF), Igwidasen Fault (SF), Adam Aghassaliyat Fault (ALF), Sebkhat Aguerj Fault (SAF), Bougouffa Fault (BF), and Sabkhat Tidzit Fault (STF). In addition to these dominant NE–SW to NNE–SSW trends, E–W-oriented structures are also recognized and locally crosscut the massif. In the eastern sector, major faults separate the two gneissified granite units and mark the eastern boundary of the complex. Other structures control the east-to-west alternation of gneissic gabbro, schist, and metabasite units. These fault systems likely played a major role in magma emplacement, tectonic segmentation, and subsequent deformation of the ASMC. Their geometry and distribution indicate a polyphase tectonic evolution linked to repeated reactivation of inherited crustal structures.

### Processing and interpretation of gamma spectrometry data

#### Contribution of radiometry

The analysis of gamma spectrometry data aims to locate anomalies, that could be correlated with lithological units and mineralization. It is more effective in regions controlled by advanced intrusion, where deliberations of radioactive components are generally more significant and revealed in a wide range of geological contexts. The texture of radiometric contour lines, representing the signatures, could be helpful in interpreting surface geology, including the lithology and the structure architecture^6,69,78^.

**Fig. 13:**
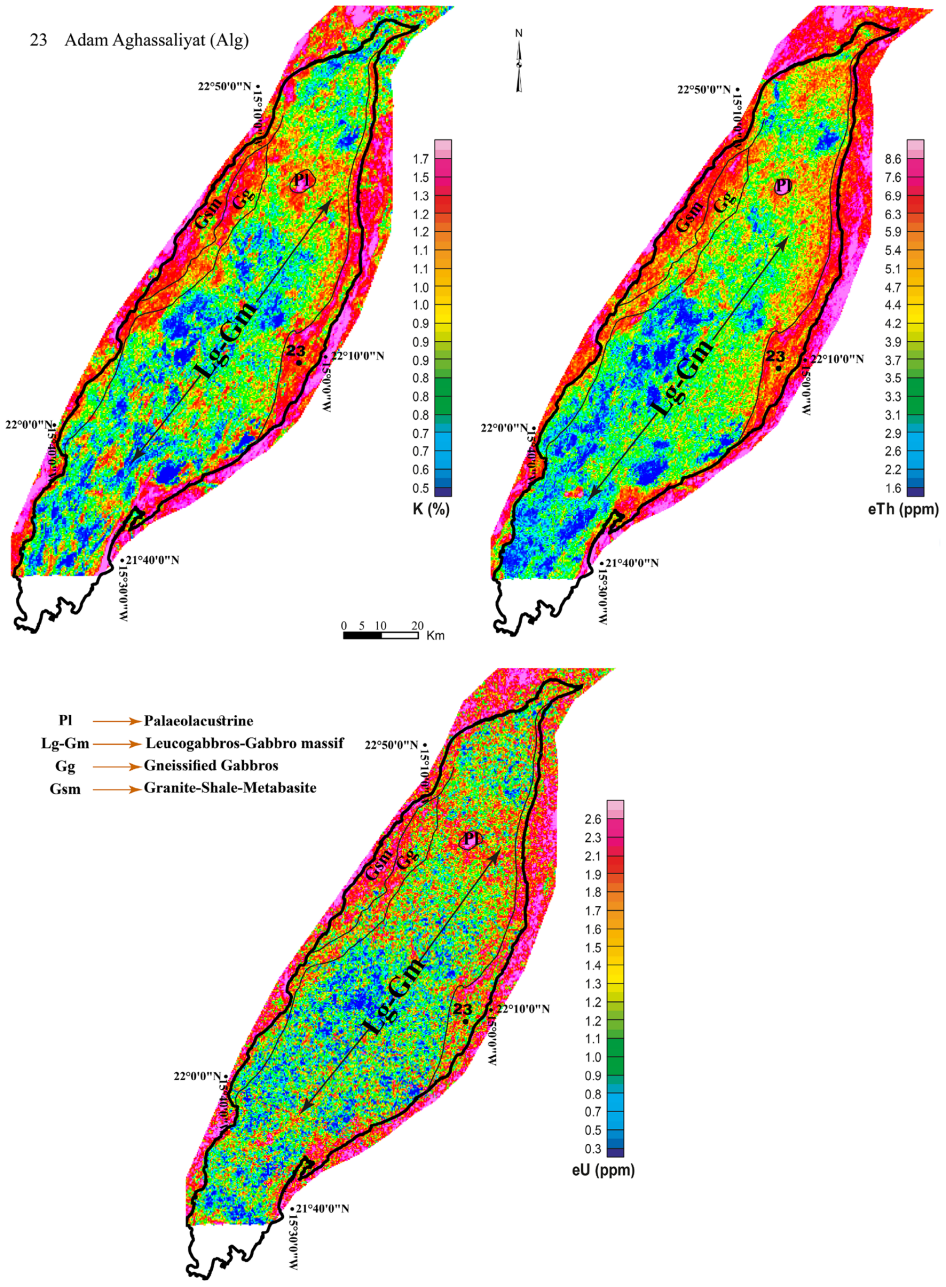
Maps of natural gamma spectrometry showing the distribution of Potassium (K%), eTh (ppm), and eU (ppm) in the ASMC.

The resulting radiometric maps provide a synthetic view of variations within geological formations based on the radiometric indices of different rock units (Fig. 13). The potassium map indicates concentrations ranging from 0.5 to 1.9% across the study area. Equivalent thorium values vary from 1.6 to 9.7 ppm, whereas equivalent uranium concentrations range between 0.2 and 2.9 ppm. The highest radioelement values (> 1.9% K, 9.7 ppm eTh, and 2.9 ppm eU) are mainly concentrated in the eastern and western sectors, as well as within the two palaeolacustrine zones identified on the ASMC map. In the eastern part of the study area (Lghassaliat), these high values are primarily associated with gneiss-marble formations, while in the western sector they correspond to granitic rocks intruded by basic dykes. Intermediate radiometric values are observed mainly in the central part of the map (0.8–1% K, 3–4.3 ppm eTh, and 0.9–1.4 ppm eU), corresponding to igneous rocks such as leucogabbro and microgabbro. A north–south-trending zone at Tachanit is characterized by shale-metabasite and gneissified gabbro. Elevated radiometric responses (1% K, 4.9 ppm eTh, and 2.9 ppm eU) are also recorded along east–west-oriented fault and fracture zones.

##### Potassium distribution derived from K/eTh

**Fig. 14:**
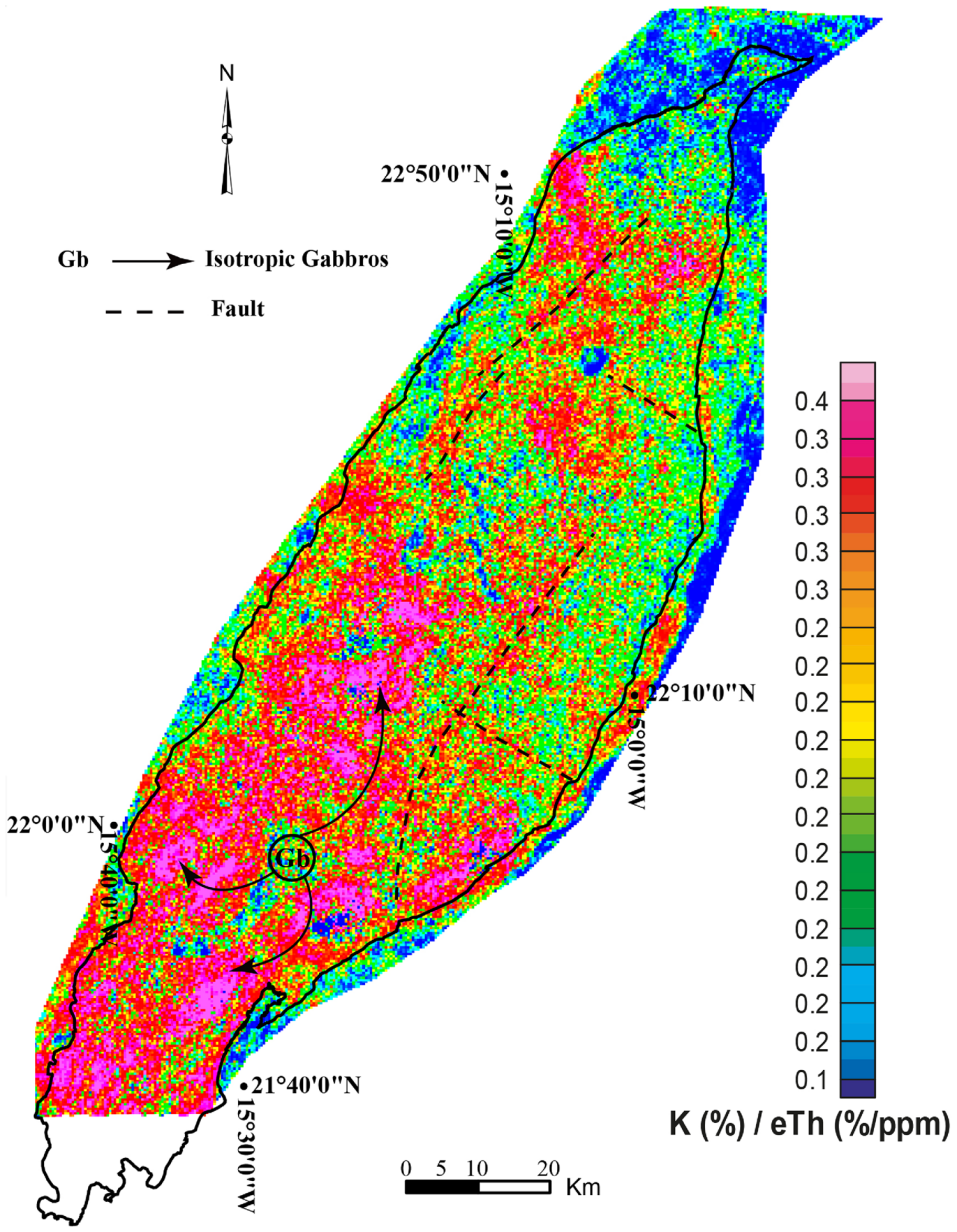
Concentrations of the Potassium-to-eThorium Ratio (K/eTh) in the study area.

The K/eTh ratio map (Fig. 14) shows values ranging from approximately 0.1 to 0.5 across the entire study area. The lowest values (~ 0.1) are mainly concentrated in the northern and western parts of the massif, where they define the background radiometric signatures. Intermediate values appear in the transition zones between the main lithological domains. The highest values (~ 0.5) are spatially associated with gabbroic units and coincide with the distribution of mafic intrusions. The spatial distribution indicates a systematic decrease in the K/eTh ratio toward the northern and western sectors. Conversely, relatively enriched ratios are restricted to mafic lithologies.

The distribution is heterogeneous and reflects distinct radiometric domains within the massif. These variations are consistent with the lithological boundaries mapped in the study area. Localized contrasts between adjacent units are observed, particularly along contacts (e.g., the Imakrane Labyad Fault (ILF)). The anomalies are controlled by structure and lithology rather than distributed randomly. Overall, the K/eTh ratio map highlights a consistent geochemical signature linked to variations in rock composition.

#### Composite image of radioelements

The highest equivalent uranium concentrations are found in bright white areas, which also show the highest potassium (%) and equivalent thorium concentrations. The Lghassalyat unit to the east and in the western part of the massif, as well as the Paleolake in the centre of the massif in the northern part, are closely associated with bright red spots that indicate the highest concentrations of eU/ eTh. The regions’ lowest concentrations of uranium and thorium are indicated by green and blue points.

**Fig. 15:**
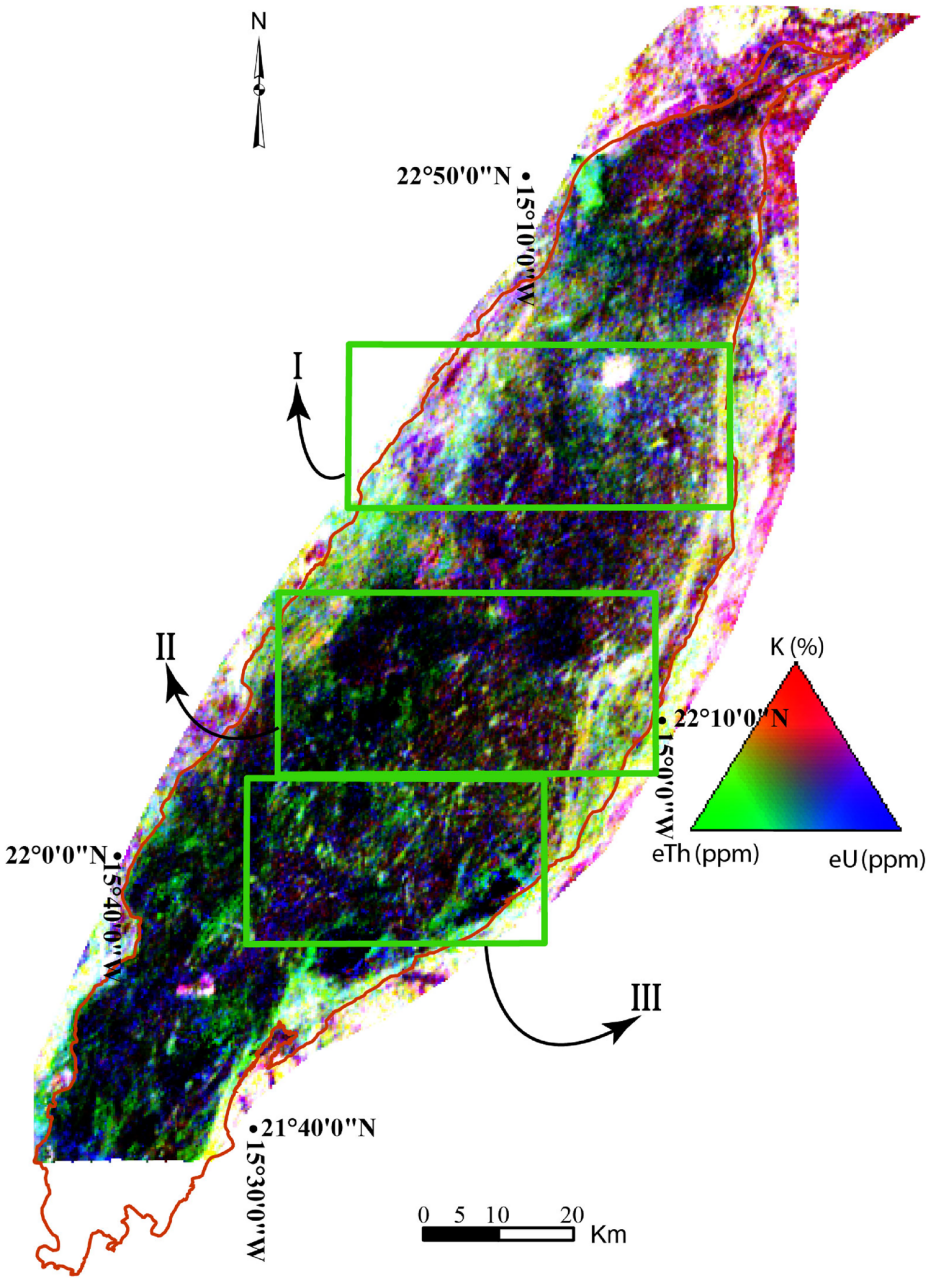
Composite image of radioelements in the ASMC. Zones I, II and III indicate location of detailed zoomed-in views presented in the Fig. 15.

Potassium (%), equivalent uranium (eU, ppm), and equivalent thorium (eTh, ppm) concentrations vary with different types of rocks. As a result, regions of consistent lithology and contacts between various lithologies can be found using concentrations obtained from gamma spectrometry data. The composite image of radioelements (Fig. 15) in the study area highlights the spatial variations in K%, eTh, and eU distributions.

Radioelement concentrations primarily reflect lithological variations. It has been observed that the brightest areas are clearly correlated with granitic rocks to the west, the Ghassalyat unit to the east, and the Quaternary in the central part of the massif to the north, which are usually distinguished by their elemental differences and robust radio-spectrometric responses. In the meantime, low radioelement concentrations are visible in dark areas of the ternary composite image, indicating weakly radioactive rocks. Another notable observation is the green color representing equivalent uranium concentration.

## Discussion

### New insights into lithological distribution and structural framework in geological mapping

The areas showing relative potassium enrichment were identified using the K/eTh ratio (Fig. 14). This parameter is widely used to identify areas affected by hydrothermal weathering and mineralization processes^66,79–82^. During fluid-rock interactions, potassium is generally more mobile than thorium, which remains relatively stable.

In weakly altered rocks, the K/eTh ratio generally remains relatively constant, with values typically ranging between 0.17 and 0.2^83^. Values exceeding this range may therefore reflect a potassium influx associated with hydrothermal alteration or potential magmatic-hydrothermal mineralization.

The resulting map highlights several areas characterized by potassium enrichment and relative thorium depletion. The high ratios observed (0.3–0.5) are consistent with active or ancient hydrothermal processes. Similar patterns, marked by an increase in potassium accompanied by a decrease in thorium, have been reported in various mineralized systems^84^. Consequently, the spatial distribution of the K/eTh ratio serves as a useful indicator for locating hydrothermally altered zones and targeting areas favorable for mineralization.

**Fig. 16:**
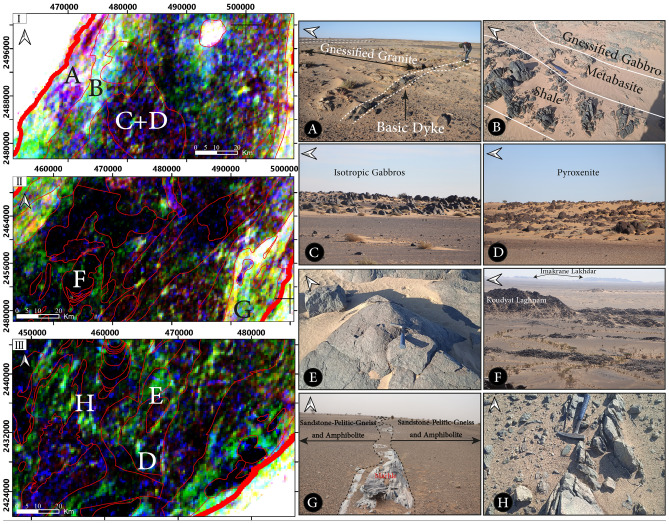
Zoom in composite image of radioelements in the study area with field photographs. Field photographs showing different lithological units of the study area. **A**) Showing the basic dyke which crosses the Gniessified granite. **B**) The Shale-Metabasite and Gneissified Gabbro is the host rock. **C**)The Isotropic Gabbro in the Gleybat Morwaguen and Gleyb Oum Lhyad **D**) The Pyroxenite in the Gleybat Morwaguen and Gleyb Oum Lhyad. **E**) Granite of the Gleybat Al Hay Aç-Camat. **F**) Panoramic view showing the isotropic gabbros of the Koudyat Lghnam. **G**) Showing the alternating of Sandstone—Pelitic—Gneiss—Amphibolite and Marble in the Adam Alghassaliyat unit. H) Showing the Foliated Leucogabbro.

Figure 15 presents the radioelement concentrations map, showing significant radiometric contrasts that reflect lithological variability across the study area. High radio-spectrometric responses are mainly observed in Adam Jadiria (eastern sector) and Adam Elghassaliat (western margin of the massif). These anomalies are interpreted as corresponding to gneissified granite in the eastern and western boundaries of the massif (Fig. 16A).

A north–south-oriented zone of Tachanit is characterized by a lithological assemblage including Shale-Metabasite and Gneissified gabbro (Fig. 16B), Gneiss-Marble, and post-tectonic Mafic dykes with Sandstone-Pelitic-Metabasite (Adam ElGassaliat) (Fig. 16D). A high range of radio-spectrometric responses is associated with Gleybat Morwaguen / Oum Lahyad, which formed by the alternation of pyroxenite and massive gabbro (Fig. 16C and D) and its adjacent zones located around the central and southern regions of the massif, composed of large mass or laccoliths (Fig. 16F). An intermediate range of radio-spectrometric responses is evident in the central parts on this map, composed of igneous rocks such as leucogabbro (Fig. 16E) and foliated leucogabbro (Fig. 16H).

**Fig. 17:**
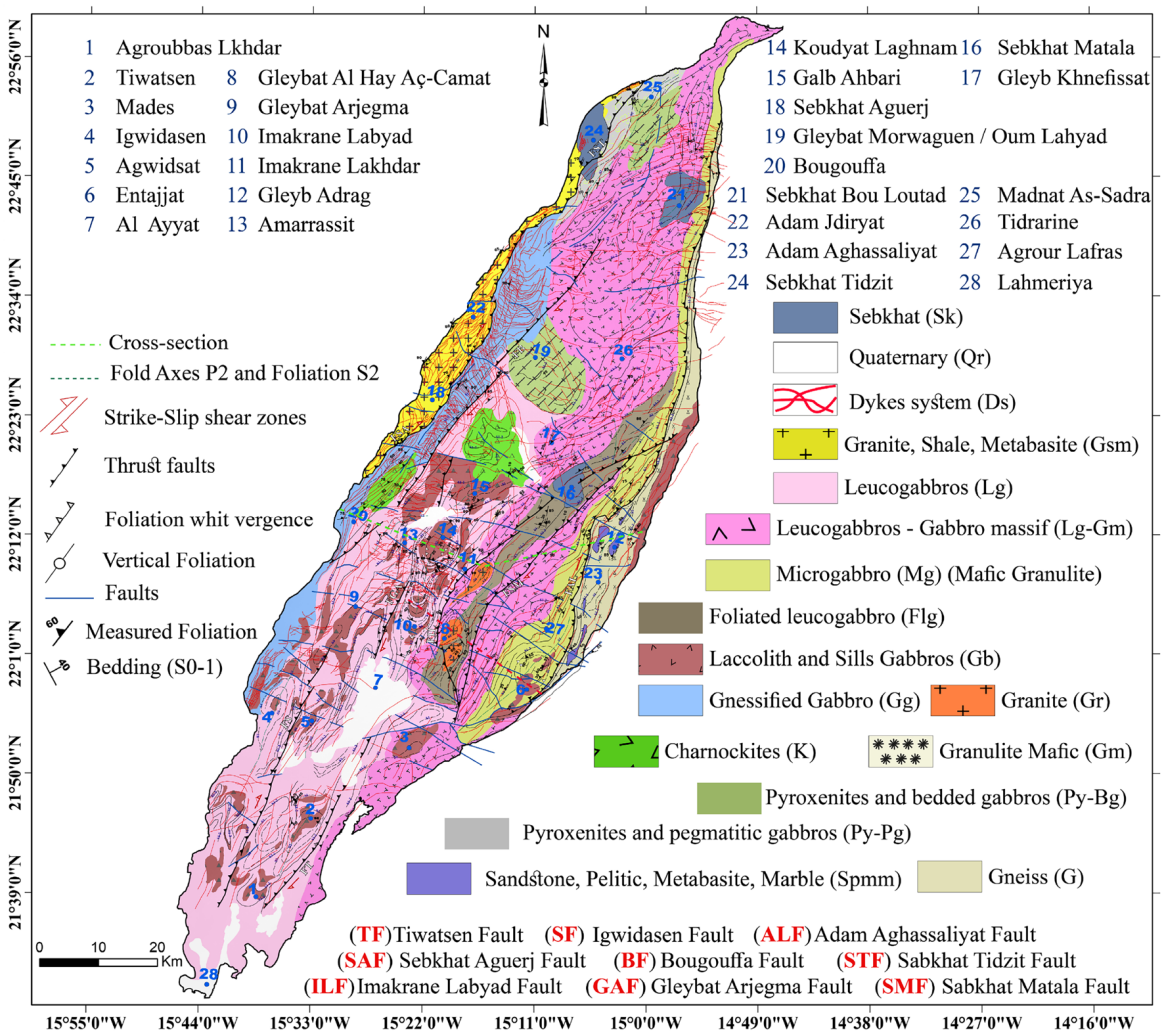
Lithological map of Adrar Souttouf mafic complex (Oulad Dlim Massif, Morocco) modified^8^,El Haous et al., Under Review).

**Fig. 18:**
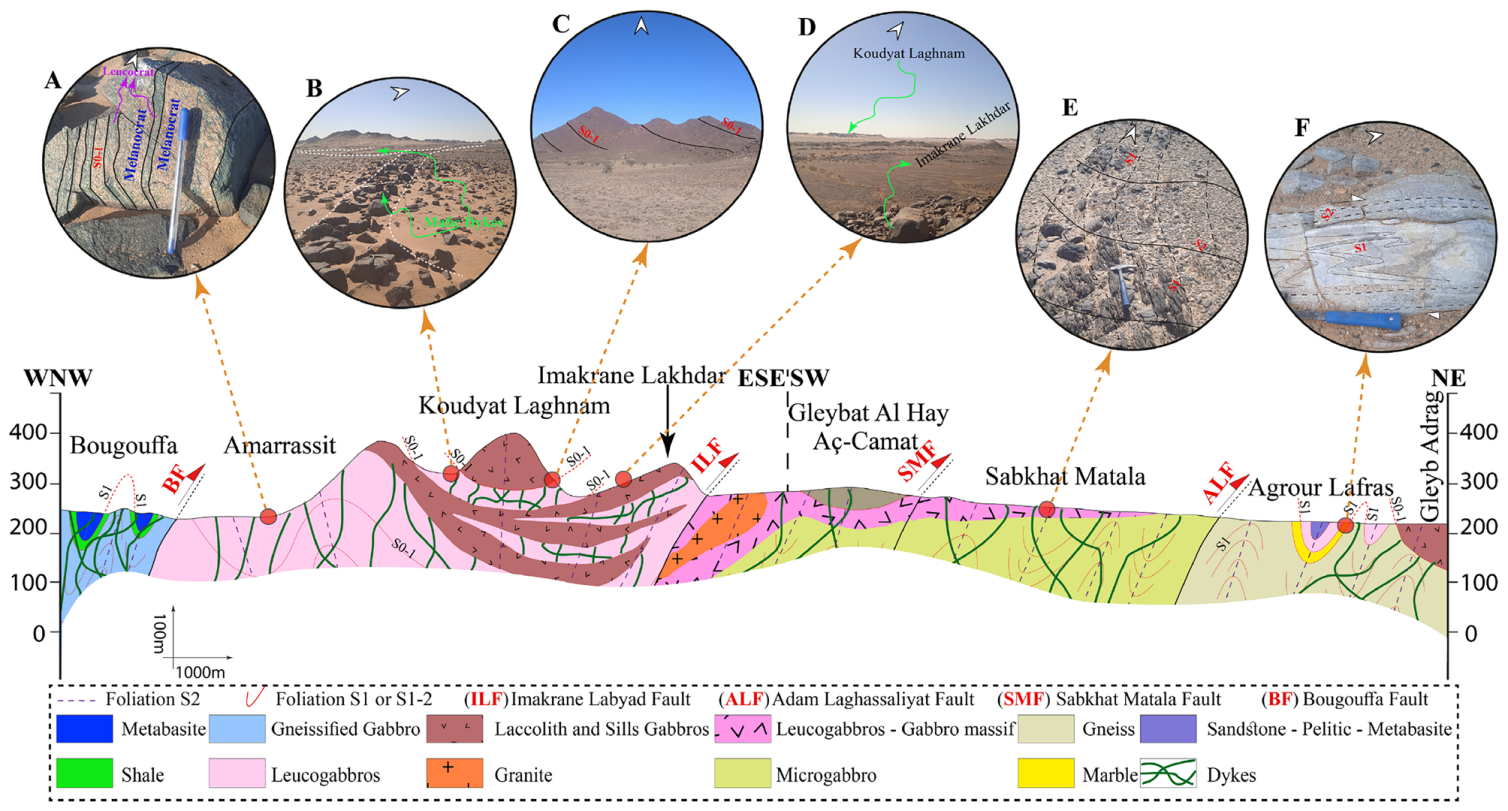
Geological cross-sections of the lithological facies of the ASMC and brittle/ductile structure. **A**) Leucogabbros are characterized by mineral alignment and compositional banding, defining alternating leucocratic and melanocratic zones. **B**) Show a dyke network acting as feeder systems that connect isotropic gabbroic laccoliths. **C**) At Koudyat Laghnam, gabbroic intrusions locally display stratiform geometries, interpreted as laccolith-like bodies emplaced within the host sequence. **D**) Spatial relationship between isotropic gabbros, which occur as massive bodies, and surrounding leucogabbros, with dyke systems locally linking and feeding these intrusive units. **E**) Leucogabbro–gabbro massif show that the P2 deformation phase overprints the earlier P1 structures, generating a new S2 foliation. This foliation locally folds and cross-cuts both S1 and the original magmatic layering (S0–1), reflecting a polyphase deformation history. **F**) Agrour Lafras area show that the marble locally preserves the S1 foliation, which is progressively transposed by the S2 fabric under a dextral shear regime.

A detailed lithological map of the study area (Fig. 17) has been produced by analysing the results of processing images and validating them through fieldwork from this study (Fig. 18) and using satellite imagery^8^ and the present study). This map includes Quaternary terrains, foliated leucogabbro (Fig. 18A), leucogabbro-gabbro massif (Fig. 18E), alternating pyroxenite-massive gabbro, granite, isotropic gabbro (Fig. 18C and D), and pegmatitic gabbro-pyroxenite in the western part of Sebkhat Tidzit, and pyroxenite-pyroxenite. Additionally, there are two units that are cut across from different directions by acidic and mafic dykes: gneissified granite at the eastern end of the massif, which is traversed by acidic and mafic dykes, and gneissic gabbro, which alternates between schist and metabasite and is traversed by mafic dykes. Alternating gneiss, marble, amphibolites, sandstone, pelitics, and microgabbro to the west of the latter form the Adam Alghassaliyat unit, another very different lithological unit. Structurally, the massif is characterized by large NE-SW to NNE-SSW lineaments (Fig. 12). This network of faults belongs to a large, well-defined shear zone composed of the: Imakrane Labyad Fault (ILF), Gleybat Arjegma Fault (GAF), Sebkhat Matala Fault (SMF), Tiwatsen Fault (TF), Igwidasen Fault (SF), Adam Aghassaliyat Fault (ALF), Sebkhat Aguerj Fault (SAF), Bougouffa Fault (BF), and the Sebkhat Tidzit Fault (STF).

In order to trace the continuity of the various geological formations that are significant to the evolution of the geology, metamorphism, and tectonics of the Adrar Souttouf mafic complex (Oulad Dlim massif, Southernmost Morocco), this lithological map of the Adrar Souttouf massif will be a valuable reference for future geological studies in this area.

**Fig. 19:**
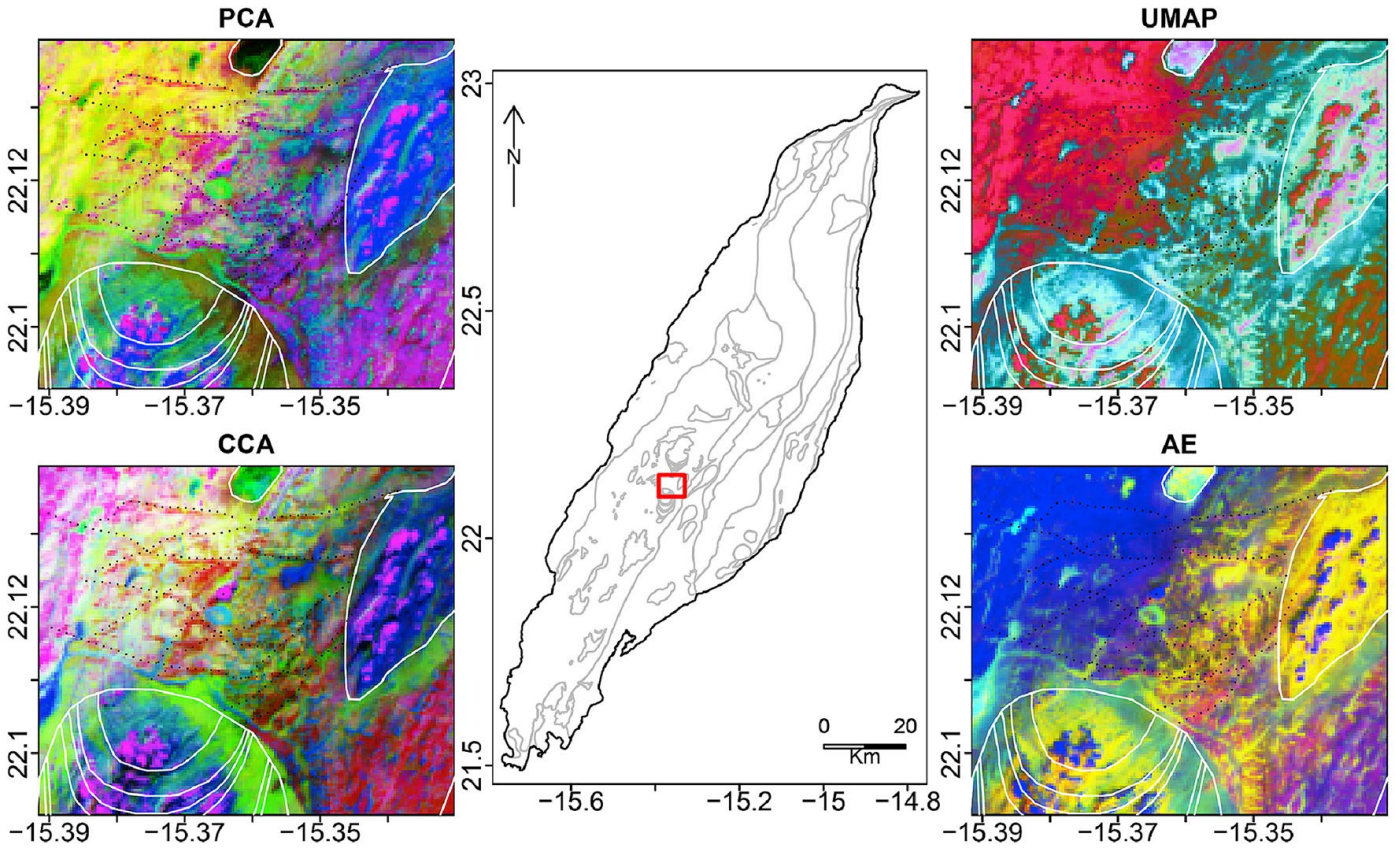
Feeder dyke’s system connecting isotropic gabbro laccoliths^8^.

Primarily to our previous research^8^, linear approaches, including principal component analysis (PCA) and canonical correspondence analysis (CCA) show superior separation of local detailed patterns, while nonlinear methods including Uniform Manifold Approximation and Projection (UMAP) and autoencoder (AE) show significant capabilities in detecting global patterns. El-Haous et al.^13^, carried out detailed mapping using long-term composite bare-earth image data and machine learning for lithological mapping. Thus, in terms of global pattern separation, UMAP performed better than other methods. These findings are in line with recent research showing the value of sophisticated analytical methods and multi-source remote sensing for mineral exploration and structural and alteration mapping^19,85,86^. Lithological mapping indicates that the ASMC is predominantly composed of mafic lithologies, including foliated leucogabbro, isotropic gabbro, pegmatitic gabbro, pyroxenite, and laccolithic intrusions, which are interconnected by a feeder dyke system (Fig. 19). To the east, the complex is bounded by the Al Ghassaliyat unit, interpreted as the country rock into which the mafic complex was emplaced.

Using very high-resolution satellite imagery combined with a composite magnetic map derived from multiple magnetic derivative products, linear magnetic anomalies were identified across the study area^13,45,46^ and the present study. The interpreted lineaments are consistent with previously mapped dyke’s networks from field studies, supporting their interpretation as magmatic dykes. The results indicate the presence of multiple generations of dykes within the ASMC, with two principal sets distinguished: one in the western sector cutting gneissified granite, shale-metabasalt, and gneissified gabbro, and another corresponding to feeder dykes associated with laccolithic gabbros distributed across the complex (Fig. 18B and D).

Structural interpretation, combined with field observations and foliation trajectory analysis, which are represented in the geological cross-sections (Fig. 18), suggests two major ductile deformation phases (P1 and P2), responsible for the development of S1 and S2 foliations, with S1 subsequently refolded during P2. The presence of E–W trending faults and intersecting dykes provides additional constraints on the internal architecture of the massif. Overall, a NE–SW to NNE–SSW lineament network defines the main structural framework of the ASMC, including several major fault systems (Figs. 12 and 18). This network of major faults generally forms dextral shear corridors, for example, the long Gleybat Al Hay Aç-Camat shear corridor defined by the ILF and SMF (Fig. 18).

### Geodynamic and geotectonic implications

In this present study the structural information is well presented in Sentinel 2A imagery (Fig. 7B), Sentinel-1 radar (Fig. 12), with most faults displaying strike-slip shear. The most important trends of these faults are E-W and NNE-SSW, while still distinguishing the isotropic basic gabbro caurs very well. High reflectance is added in this composition for gabbro, making this NNE-SSW unit appear blue (Fig. 7B). The complex is a polyphase magma chamber, indicating prolonged and multi-stage magmatic activity, according to the lithological mapping of the ASMC^8^. The observed compositional diversity—from mafic to felsic intrusions—indicates that magma has been injected repeatedly, which is more common in intracontinental rift settings than in island arc environments (El Haous et al.^87^ and the present study).

The significant lack of mantle-derived rocks, like peridotites, which were previously noted by^91^, and the absence of pillow-lava basalts, which are typically suggestive of submarine volcanic processes, lend credence to this rift-related interpretation. The model of an island arc forming within the West African Neoproterozoic Ocean (WANO), put forth by Villeneuve et al.^37^ and Gärtner et al.^88^, is incompatible with these lithological and petrological features. Thus, rift-driven magmatism and crustal extension serve as the primary mechanisms of the ASMC’s formation in the new interpretation, which highlights the dominance of crustal processes in its evolution. This perspective supports the notion that intracontinental geodynamic settings, rather than subduction-related processes, were crucial in forming the complex, and it partially supports the findings of Bea et al.^40^ and Cambeses et al.^44^.

Despite the robustness of the integrated approach, the study is limited by the shallow penetration depth of gamma-ray spectrometry, which only reflects near-surface conditions^22,26^, as well as by the spatial resolution of remote sensing datasets, which may not capture very small-scale geological features^15,57,89^. Nevertheless, the integration of field observations with multi-source geospatial data provides a consistent qualitative framework supporting the geological interpretations presented in this work.

## Conclusion

This study provides the first attempt integrating radar, optical and airborne gamma ray spectrometry data to better characterize and update the litho-structural characteristics of the studied terrain. The study concluded that:

1. A new geological map depicting the Adrar Souttouf mafic complex (Oulad Dlim massif, southern Morocco) has been created. To create this map, radiometric data, Landsat-8 and Sentinel 2A imagery, Sentinel-1 radar, and field observations were integrated.
2. Airborne radiometric data and multispectral satellite imagery were used to characterize the lithological units of the study area. Radiometric signatures allowed the distinction of major rock types, including granitic, gabbroic, and pyroxenitic units, while RGB composites from Landsat-8 OLI and Sentinel-2A facilitated the discrimination of surface lithologies such as Quaternary deposits, sebkhas, schists, and mafic–ultramafic units.
3. The interpretation of RTC-processed Sentinel-1 radar data, supported by optical imagery and lithological relationships, allowed the extraction of major fault systems affecting the study area. The integration of remote sensing, field observations, and geophysical data provides a consistent structural framework and improves the understanding of the litho-structural organization of the Oulad Dlim Massif within the Adrar Souttouf mafic complex.
4. This study provides us with several key findings, including a more detailed geological analysis and the identification of the faults that control these facies. These findings allow us to better understand the geology of our mountain range and compare it on a regional scale. These insights can serve as a guide for future geological and mining exploration studies.

By combining these datasets, allowed a better characterization of the main deformation zones and tectonic contacts within the study area. New understanding of the tectonic development of the Ediacaran domain and its connection to the nearby Archean blocks is made possible by this improved structural framework. Complementary microstructural and microtectonic investigations are currently underway to further constrain the multi-phase tectonic evolution of the massif.

## Data Availability

The datasets used and/or analyzed during the current study are available from the corresponding author upon reasonable request.
